# Clinical validation of the diagnosis adverse location of peripherally inserted central catheter in neonatology

**DOI:** 10.1590/1980-220X-REEUSP-2025-0117en

**Published:** 2025-08-04

**Authors:** Nanete Caroline da Costa Prado, Richardson Augusto Rosendo da Silva, Harlon França de Menezes, Isabele Silva dos Santos, Rebecca Stefany da Costa Santos, Sérgio Danillo Santana de Lima Juraci, Dhyanine Morais de Lima Raimundo, Romanniny Hévillyn Silva Costa Almino

**Affiliations:** 1 Universidade Federal do Rio Grande do Norte, Programa de Pós-graduação em Enfermagem, Natal, RN, Brazil.

**Keywords:** Standardized Nursing Terminology, Nursing Diagnosis, Validation Study, Intensive Care, Neonatal, Catheterization, Central Venous

## Abstract

**Objective:**

To determine the accuracy measures of clinical indicators of Nursing Diagnosis (ND) of the International Classification of Nursing Practice (ICNP)®, Adverse Location of Peripherally Inserted Central Catheter in a Neonatal Intensive Care Unit (NICU).

**Methods:**

Diagnostic accuracy study carried out with 40 neonates admitted to a NICU in Northeastern Brazil. Data collection was performed through clinical examination and application of an institutional instrument. The clinical indicators analysis of the respective ND was performed using accuracy measures.

**Results:**

Among the study participants, the majority were female and had low birth weight. The prevalence of ND was 57.5%. The clinical indicators ‘difficulty in catheter progression’ and ‘absence of blood return’ showed high specificity values. The catheter insertion site, prematurity, and the occurrence of adverse events presented higher sensitivity values.

**Conclusion:**

Accurate diagnostic inference allows the early identification of inadequate catheter tip positioning, supporting nurses in developing precise and effective interventions to avoid complications.

## INTRODUCTION

The Neonatal Intensive Care Unit (NICU) is an environment designed to care for children up to 28 days old, providing highly complex care and specialized care in neonatology. The procedures are aimed at maintaining life, and commonly involve several invasive procedures, such as the Peripherally Inserted Central Catheter (PICC)^(1)^.

The term PICC refers to a long-term catheter used for the administration of intravenous solutions. The device has a slender and flexible design, allowing its placement through a simple puncture of a peripheral vein under direct visualization. The distal end of the catheter should be positioned in the distal third of the superior vena cava when the puncture is performed from the upper limb, and in the inferior vena cava when inserted via the lower limbs^(2,3)^.

The insertion and management of the PICC fall under the nurse’s competencies, as regulated by Resolution No. 258/2001 of the Federal Nursing Council (COFEN). This regulation establishes guidelines for the role of nursing professionals in inserting PICC in neonates, children, and adults, provided they are trained and qualified to perform the procedure^(4)^.

In the NICU, the nurse plays an essential and qualified role in the care of newborns using catheters, a role that requires specific clinical skills, updated technical knowledge, and safe decisions to minimize risks such as infections, migration of the catheter tip, obstruction, and thrombosis.

However, the use of the device is often associated with improper positioning of the distal end during insertion, which poses a risk of adverse conditions of varying severity. If not identified early, this complication may lead to corrective interventions, which also carry risks. Therefore, it is essential that healthcare professionals develop advanced clinical reasoning skills to recognize indicators of improper positioning of the peripherally inserted central catheter, minimizing its occurrence^(5,6)^.

In this scenario, the Nursing Process, based on the nurse’s critical thinking and clinical judgment, becomes essential to support care appropriately, promoting the standardization of practice by the Nursing team through uniform language. To this end, the International Classification of Nursing Practice (ICNP)^®^ provides the necessary support for nursing care by enabling the development of terminological subsets composed of statements of nursing diagnoses, outcomes, and interventions appropriate for particular areas of care^(7,8)^.

Furthermore, it contributes to a better implementation of nursing care by providing indicators and patient safety and for conducting research, since it is an international standard terminology, with a combinatorial and enumerative structure, recognized worldwide, adaptable to different environments, including pediatric and neonatal ones.

Thus, recognizing and clinically applying clinical indicators through validation studies becomes essential to ensure their effectiveness in practice and enable comparative analysis of the replication of the care model in different contexts. This process provides both internal and external validity to the study. Clinical validation involves the collection of data to verify the presence of clinical indicators associated with the statements, including the Nursing Diagnosis (ND) of improper positioning of the peripherally inserted central catheter.

A review of the state of the art on the study object revealed a lack of specific research evaluating clinical indicators for nursing diagnoses in newborns with PICC, justifying the need for the present study^(8,9)^. Accuracy studies aim to identify which indicator best predicts the occurrence of the ND, guiding the selection of the most appropriate interventions for nursing care^(10)^. Conducting such studies is crucial to ensure that the ICNP® nursing diagnosis taxonomy is continuously reviewed and updated, impacting nursing practice and enhancing patient safety.

It is expected that this study will assist nurses working in NICU in identifying the indicators that best predict the occurrence of the diagnosis in question. Thus, it will contribute to a more precise inference, guiding the selection and implementation of appropriate nursing care interventions for this population, reinforcing the relevance of the research.

Given the problem, the following questions were raised: what is the prevalence of the ND adverse location of the peripherally inserted central catheter in newborns admitted to the NICU? How accurate are its clinical indicators?

Therefore, the objective of the study in question was to determine the accuracy measures of the clinical indicators of the ICNP^®^ ND adverse location of peripherally inserted central catheter in neonates admitted to the NICU.

## METHOD

### Design of Study

This research refers to a section of a larger project, where the objective was to clinically validate the clinical indicators of nursing diagnoses of a terminological subset of the ICNP® for newborns with PICC in a NICU^(8,9)^. Based on its application in clinical practice, the need for an update and mapping with the Systematized Nomenclature of Medicine Clinical Terms (SNOMED CT) was identified. Accordingly, a new content validation was conducted by experts, followed by clinical validation through diagnostic accuracy assessment.

Given the extensive number of diagnoses present in the referenced subset, this manuscript presents only the accuracy of the nursing diagnosis of improper positioning of the peripherally inserted central catheter, as it was the most prevalent during clinical validation. This is a diagnostic accuracy study with an analytical design and a quantitative approach. The report was prepared following the guidelines of the Standards for Reporting Studies of Diagnostic Accuracy (STARD) initiative, ensuring clarity, transparency, and quality in the presentation of results^(11)^.

The clinical indicators of the referenced nursing diagnosis were measured, including difficulty in catheter progression, absence of blood return, catheter insertion site, prematurity, and occurrence of adverse events, according to the following operational definitions:

- Difficulty in catheter progression: characterized by the inability to advance the catheter through the introducer or peripheral circulation, the impossibility of reaching the previously measured distance, or the difficulty in transitioning the catheter from peripheral to central circulation;
- Absence of blood return: occurs when the catheter inserter does not obtain a return of blood flow immediately after insertion;
- Catheter insertion site: refers to the site chosen by the inserter for catheter introduction and advancement;
- Prematurity: defined as the birth of a newborn before the completion of 37 full weeks of gestation, meaning a gestational age of less than 37 weeks;
- Occurrence of adverse events: complications that may arise during or after catheter use, compromising the safety and effectiveness of intravenous treatment^(12,13)^.

### Local

The study was carried out in a NICU in the Brazilian Northeast, linked to the Brazilian Public Health System.

### Population

The study population consisted of 40 neonates admitted to the NICU and using the PICC in a critical care maternity hospital in Northeastern Brazil. In addition to the newborns, other participants took part in the study, including four nurses who acted as evaluators of the case studies.

### Selection Criteria

Newborns were selected by convenience and consecutively, with the following eligible inclusion criteria: newborns admitted to the NICU using PICC and who remained in the sector until the end of treatment. The exclusion criteria adopted were: the transfer of the newborn to another institution and newborns whose mothers were adolescents. The exclusion of adolescent mothers was based on ethical guidelines governing research involving human subjects (Resolution CNS 466/12), as minors, including adolescents, do not have full civil capacity and therefore require a legal guardian to sign the Informed Consent Form (ICF). It is noteworthy that the ICF was read, explained, and all doubts were clarified for the parents or guardians of the newborn.

Regarding the selection of nurses, they possessed knowledge and experience in neonatal intensive care and were trained in the insertion of the PICC, while also meeting all inclusion criteria. The inclusion criteria were: having a specialty in neonatology and at least five years of clinical care experience in the field; having completed a certification course in catheter insertion; and having practical experience of at least two years in PICC insertion. Professionals on leave or vacation during the data collection period were considered exclusion criteria.

Access to specialist nurses occurred in person at the hospital where the research was conducted. During the initial contact, an invitation was extended, and the study’s objectives, risks, and benefits were explained. Subsequently, those who agreed to participate signed the Informed Consent Form (ICF).

### Sample Definition

To establish the sample of newborns, the formula for calculating samples for finite populations was used, to provide the efficiency and effectiveness of this research in an impartial, safe, and scientific manner. The sample size was calculated using the following procedure: a significance level (a) of 5% and a margin of error of 0.05 were assigned. When the proportion was unknown, p = 0.5 was adopted, considering maximum variance. For the selection of study specialists, intentional non-probabilistic sampling was used.

### Data Collection

To achieve the objective, the global project was operationalized according to the following phases: 1) Implementation of the Nursing Process according to the following stages: nursing assessment, ND, nursing planning, implementation and nursing evolution; 2) Preparation of case studies and statements of nursing diagnoses/outcomes, and interventions (ND/NO/NI); 3) Analysis of agreement among specialist nurses; 4) Accuracy of clinical indicators of nursing diagnoses of the terminology subset^(8)^. The data collection period was from October to December 2024.

The statements and their respective clinical indicators were tested in a neonatal population using the following accuracy measures: sensitivity, specificity, positive and negative predictive values, and positive and negative likelihood ratios.

Patient data were collected through physical examination and analysis of nursing records contained in the sample’s medical charts. Data collection and diagnostic inference were carried out by the principal researcher in collaboration with a doctoral student in Nursing.

For the study, an instrument based on Betty Neuman’s theoretical model was used, structured into the following sections: sociodemographic data, assessment, diagnosis, planning, implementation, and nursing evolution. Before the final application, a pilot test was conducted previously approved by the research ethics committee with the objective of verifying the adequacy of the instrument. Notably, participants in the pilot test were not included in the main study.

The experts received spreadsheets developed by the research duo, each corresponding to a patient and containing all the terms, ND/NO and NI according to the employed theoretical model. The spreadsheets also included complementary information on socioeconomic and clinical data, as well as relevant observations regarding the diagnostic inference process. Each expert independently evaluated the presence or absence of the ND/NO and NI in each of the received spreadsheets.

For this research, clinically validated ND/NO and NI were those identified in newborns, described in case studies, and included in the terminological subset. The agreement analysis among the nurse judges was conducted using the Kappa (k) concordance coefficient, with specialists reviewing case studies and the listed statements.

The final stage of the study involved evaluating the accuracy of the clinical indicators of the NDs within the terminological subset, aiming to determine the precision of these indicators in predicting the occurrence of the investigated diagnoses among neonates using the peripherally inserted central catheter (PICC). The study analyzed which indicator demonstrated the highest predictive accuracy for the occurrence of the investigated ND.

In this study, clinical indicators were defined based on literature that supports clinical decision-making. Given the journal’s page limitations, this article will focus exclusively on the accuracy of clinical indicators for the ICNP ND “improper positioning of the peripherally inserted central catheter,” as it was the most prevalent in the research. This approach is necessary due to the extensive number of indicators within the diagnostic subset. It is noteworthy that other results from the broader project will be published in future scientific articles.

### Data Analysis and Treatment

Data analysis was performed through the verification of the level of agreement and level of consistency (reliability) of the judges’ (specialist nurses) opinions regarding the other items of the terminological subset, with Kappa (k) coefficient of agreement^(14)^ being applied.

This index was also applied to verify inter-rater agreement for each item. The Kappa concordance coefficient assesses the proportion of agreement, ranging from “minus 1” to “plus 1”; the closer to 1, the higher the level of agreement among observers. Each judge indicated whether the item was relevant and whether the respective definitions were clear and precise, selecting one of five suggested responses (strongly agree – 1; partially agree – 2; neutral – 3; partially disagree – 4; totally disagree – 5).

Each response was assigned a score: strongly agree – 1.0; partially agree – 0.75; neutral – 0.50; partially disagree – 0.25; totally disagree – 0.0. This criterion was used for both relevance and applicability of each item. An acceptance criterion of agreement ≥ 0.80 among judges was established, which was considered a good level.

The agreement among the four specialist nurses was evaluated using the Kappa coefficient (k), classified as follows: poor (0 to 0.19); fair (0.20 to 0.39); moderate (0.40 to 0.59); substantial (0.60 to 0.79); near perfect (0.80 to 0.99); and perfect (equal to 1)^(15)^.

The nonparametric Kruskal-Wallis test was used to verify whether there were differences in the means of the specialist nurses’ assessments. The significance level adopted was p ≤ 0.05; therefore, a difference between the means was considered statistically significant when the p value was less than or equal to this limit. To assess the reliability and applicability of the subset in different contexts, the Intraclass Correlation Coefficient (ICC) was used, where ICC ≥ 0.75 indicates excellent reliability; ICC between 0.40 and 0.75 denotes satisfactory reliability; and ICC < 0.40 is considered poor^(14,15)^.

The data related to the ND and NO were analyzed using descriptive statistics, considering absolute and relative frequencies. Information about NI was presented in a grouped format, following the theoretical model categories and discussed according to their frequencies. Subsequently, the data were organized in an electronic spreadsheet, and after double-entry verification, they were exported to the Statistical Package for Social Science (SPSS), version 20.0 for Windows. After coding and tabulation, the data were analyzed through reflective reading and statistical methods, applying the Kappa test using the Online Kappa Calculator.

Based on the study’s objectives and nature, the following hypotheses were formulated: Null Hypothesis (H_0_): The clinical indicators of the ND “improper positioning of the peripherally inserted central catheter” do not present significant accuracy measures for diagnostic inference in neonates admitted to the NICU. Alternative Hypothesis (H_1_): The clinical indicators of the ND “improper positioning of the peripherally inserted central catheter” present significant accuracy measures for diagnostic inference in neonates admitted to the NICU.

The clinical validation analysis was conducted in two phases. The first phase involved characterizing the population using descriptive statistics. In the second phase, the accuracy measures of the clinical indicators in relation to the ND were examined. These measures were calculated based on diagnostic inference of the presence of the ND, establishing a cutoff point of 50%. To assess associations between qualitative variables and clinical indicators, the Chi-Square Test or Fisher’s Exact Test was applied.

### Ethical Aspects

The study was approved by the Research Ethics Committee with opinion number 7.171.001 of 2024 and complied with the ethical principles of Resolution No. 466, of December 12, 2012, of the National Health Council, which discusses research involving human beings.

## RESULTS

Forty newborns participated in this study, of which 65% (26) were women, with a predominance of babies born by cesarean section (70% – 28), low birth weight (65% – 26), and with an average APGAR of 6.46 and 8.52 in the first and fifth minute of life, respectively. The average length of stay of PICC was 10.23 days.

The main indications for catheter insertion were antibiotic therapy (80%) and parenteral nutrition (65%). Regarding the clinical diagnosis, respiratory conditions (75%) and prematurity (70%) were the most prevalent. The type of catheter used was single-lumen silicone. In terms of insertion sites, the upper limbs were the most common (55%). All catheters were inserted by nurses.

Regarding the prevalence of clinical indicators for the ICNP® nursing diagnosis of improper positioning of the peripherally inserted central catheter, the following were observed: “difficulty in catheter progression” (57.5%), “absence of blood return” (55%),“catheter insertion site (upper limbs)” (55%), “occurrence of adverse events” (50%), “prematurity (< 37 weeks)” (47.5%), “newborn’s weight” (45%), “presence of an umbilical venous catheter” (32.5%), “presence of another peripherally inserted catheter” (27.5%), “congenital malformation” (7.5%).

Table 1 presents the prevalence ratios of the clinical indicators analyzed according to the occurrence of the ND Adverse Location of the Peripherally Inserted Central Catheter in neonates admitted to the NICU. The data highlight the clinical indicators that showed a statistically significant association with the diagnosis, as well as their prevalence ratios and confidence intervals. These findings emphasize the most relevant factors for diagnostic inference, contributing to a more precise and well-founded analysis.

**Table 1:** Prevalence ratios of clinical indicators according to the occurrence of the ND adverse location of peripherally inserted central catheter in newborns in the NICU – Natal, RN, Brazil, 2024.

Variables	ND adverse location of peripherally inserted central catheter						
Clinical indicators	Present		Absent		Total		Statistics
	N	%	N	%	N	%	
Difficulty in catheter progression							
Present	23.0	57.5	0.0	0.0	23.0	57.5	*p* = 0.001*
Absent	0.0	0.0	17.0	42.5	17.0	42.5	PR^†^ = 4.98^†^
Total	23.0	57.5	17.0	42.5	40.0	100.0	95%^‡^CI: 3.09–5.88^‡^
Absence of blood return							
Present	21.0	52.5	1.0	2.5	22.0	55.0	*p* = 0.001*
Absent	4.0	10.0	14.0	35.0	18.0	45.0	PR^†^ = 4.02^†^
Total	23.0	57.5	17.0	42.5	40.0	100.0	95%^‡^CI: 2.71–5.17^‡^
Catheter insertion site (upper limbs)							
Present	19.0	47.5	3.0	7.5	22.0	55.0	*p* = 0.038*
Absent	4.0	10.0	14.0	35.0	18.0	45.0	PR^†^ = 3.95^†^
Total	23.0	57.5	17.0	42.5	40.0	100.0	95%^‡^CI: 2.15–4.80^‡^
Occurrence of adverse events							
Present	18.0	45.0	2.0	5.0	20.0	50.0	*p* = 0.002*
Absent	5.0	12.5	15.0	37.5	20.0	50.0	PR^†^ = 2.98^†^
Total	23.00	57.5	17.0	42.5	40.0	100.0	95%^‡^CI: 1.84–3.56^‡^
Prematurity (< 37 weeks)							
Present	16.0	40.0	3.0	7.5	19.0	47.5	*p* = 0.012*
Absent	7.0	17.5	14.0	35.0	21.0	52.5	PR^†^ = 2.92^†^
Total	23.0	57.5	17.0	42.5	40.0	100.0	95%^‡^CI: 1.69–3.28^‡^
Newborn weight							
Present	11.0	27.5	7.0	17.5	18.0	45.0	*p* = 0.332^§^
Absent	12.0	30.0	10.0	25.0	22.0	55.0	PR^†^ = 1.48^†^
Total	23.0	57.5	17.0	42.5	40.0	100.0	95%^‡^CI: 0.86–1.98^‡^
Presence of Umbilical Venous Catheter							
Present	9.0	22.5	4.0	10.0	13.0	32.5	*p* = 0.217*
Absent	14.0	35.0	13.0	32.5	27.0	67.5	PR^†^ = 1.46^†^
Total	23.0	57.5	17.0	42.5	40.0	100.0	95%^‡^CI: 0.71–1.92^‡^
Presence of another Peripherally Inserted Venous Catheter							
Present	8.0	20.0	3.0	7.5	11.0	27.5	*p* = 0.402*
Absent	15.0	37.5	14.0	35.0	29.0	72.5	PR^†^ = 1.34^†^
Total	23.0	57.5	17.0	42.5	40.0	100.0	95%^‡^CI: 0.97–2.28^‡^
Congenital malformation							
Present	2.0	5.0	1.0	2.5	3.0	7.5	*p* = 0.619*
Absent	21.0	52.5	16.0	40.0	37.0	92.5	PR^†^ = 1.21^†^
Total	23.0	57.5	17.0	42.5	40.0	100.0	95%^‡^CI: 0.82–2.12^‡^

*Fisher’s exact test;

^†^PR = prevalence ratio;

^‡^CI = 95% confidence interval;

^§^Pearson’s chi-square test; p < 0.05.

Based on Table 1, the clinical indicators “Difficulty in catheter progression,” “Absence of blood return,” “Catheter insertion site (upper limbs),” “Occurrence of adverse events,” and “Prematurity (< 37 weeks)” were found to be risk factors for the studied nursing diagnosis (ND). Furthermore, an increased likelihood of newborns developing the ND “improper positioning of the peripherally inserted central catheter” was observed, with the following prevalence ratios (PR): 5 times (PR = 4.98), 4 times (PR = 4.02), 4 times (PR = 3.95), 3 times (PR = 2.98), and 3 times (PR = 2.92), respectively.

On the other hand, the indicators “Newborn weight,” “Presence of an Umbilical Venous Catheter,” “Presence of another Peripherally Inserted Catheter,” and “Congenital malformation” did not show a statistically significant association with the ND “improper positioning of the peripherally inserted central catheter.”

Table 2 details the accuracy measures of clinical indicators related to the ND Adverse Location of Peripherally Inserted Central Catheter in newborns admitted to the Neonatal Intensive Care Unit.

**Table 2:** Accuracy measures of clinical indicators of ND, adverse location of peripherally inserted central catheter in newborns in the Neonatal Intensive Care Unit – Natal, RN, Brazil, 2024.

Clinical indicators	Se*	Sp^†^	PPV^‡^	NPV^§^	PLR^||^ (95% CI)	NLR^¶^ (95% CI)	DOR** (95% CI)
Difficulty in catheter progression	100.0	45.28	100.0	16.97	9.00 (7.87–11.67)	0.67 (0.31–0.91)	8.92 (6.91–10.68)
Absence of blood return	98.34	51.10	98.41	29.55	6.31 (4.24–8.12)	0.62 (0.25–0.87)	6.21 (4.16–8.74)
Catheter insertion site (upper limbs)	57.31	92.30	97.12	23.45	4.84 (2.14–6.92)	0.53 (0.41–0.77)	5.00 (3.24–7.61)
Occurrence of adverse events	48.87	89.93	91.39	19.38	3.33 (1.18–5.73)	0.30 (0.19–0.64)	4.93 (2.84–6.75)
Prematurity (< 37 weeks)	54.21	94.85	99.82	22.31	3.46 (1.28–5.98)	0.76 (0.62–0.83)	6.90 (4.55–8.65)
Newborn weight	13.26	54.58	97.50	28.44	1.26 (0.02–2.63)	0.88 (0.53–1.69)	2.76 (0.95–4.08)
Presence of Umbilical Venous Catheter	31.16	64.07	57.41	26.84	1.46 (0.65–3.86)	0.65 (0.21–1.10)	1.93 (0.89–4.32)
Presence of another Peripherally Inserted Venous Catheter	33.08	64.13	96.30	22.17	1.98 (0.85–4.12)	0.82 (0.55–1.17)	2.11 (0.91–4.85)
Congenital malformation	3.29	44.55	97.50	18.42	1.37 (0.14–3.29)	0.55 (0.13–1.12)	1.41 (0.67–3.28)

*Se = sensitivity;

^†^Sp = specificity;

^‡^PPV = positive predictive value;

^§^NPV = negative predictive value;

^||^PLR = positive likelihood ratio;

^¶^NLR = negative likelihood ratio;

**DOR = Diagnostic *Odds Ratio*.

The prevalence of the ND improper positioning of the peripherally inserted central catheter was 57.5%. The clinical indicators “difficulty in catheter progression” and “absence of blood return” exhibited high specificity values, as well as statistically significant likelihood ratios and OR, suggesting they are strong indicators for confirming the presence of the diagnosis.

Additionally, the clinical indicators “catheter insertion site,” “prematurity,” and “occurrence of adverse events” demonstrated higher sensitivity values, accompanied by statistically significant likelihood ratios and ORs, indicating that these indicators have the best accuracy measures for inferring the studied ND.

## DISCUSSION

The ND Adverse Location of Peripherally Inserted Central Catheter was clinically validated by accuracy measures, corroborating the literature, which emphasizes the importance of monitoring the positioning of the catheter tip, as the incidence of poor positioning varies between 10% and 60%. Such inadequacy may result in significant adverse events, such as cardiac tamponade, pleural and pericardial effusion, infiltration, endothelial injury, among others, representing a considerable risk to patient safety^(16,17,18)^.

The occurrence of inappropriate PICC catheter positioning in newborns in intensive care is considered to be significantly more common, estimated to be approximately three times higher compared to other central venous accesses^(19)^.

It has been reported that PICC migration occurs within the first 24–72 hours after insertion^(12,20)^. Therefore, some researchers have suggested performing a repeat radiograph 24 hours after PICC insertion to identify migration, whereas others have suggested performing imaging surveillance up to 72 hours after PICC insertion^(12)^.

Regarding risk factors, the clinical indicator “difficulty in catheter progression” was considered a predictor of the ND at issue. This finding can be evidenced as inadequate progression occurs due to incorrect PICC path, resulting in catheter tangling and advancement towards collateral vessels close to the central circulation (subclavian, jugular, and axillary veins), being associated with the outcome of peripheral position^(21)^. Difficulty in PICC progression can be defined as the inability to pass the catheter through the introducer or into the peripheral circulation, the inability to pass the catheter to the previously measured distance, or the inability to pass the catheter from the peripheral circulation to the central circulation^(12)^.

In a study carried out in a NICU, difficulty in progression was observed in 63 procedures (25.8%), indicating a significant correlation with the initial position of the catheter tip. Difficulty in progression was reported in 50% of catheters with a non-central initial position and in 22% of catheters with a central position^(22)^. Another study that corroborates the findings of the present research was carried out with 141 neonates, which identified that the difficulty in progressing the PICC during insertion was associated with migration of the catheter tip (p < 0.01)^(12)^.

The “absence of blood return” immediately after catheter insertion, also called the flow and reflux test, was considered a risk factor for false PICC path and was a clinical indicator related to the ND studied, also being considered a predictor for adverse location of the peripherally inserted central catheter in neonatology. This finding corroborates the literature, which highlights that, in addition to the difficulty in advancing the catheter during insertion, impaired blood aspiration has long been reported as a predictor that poor catheter implantation may have occurred^(23)^.

A study involving 100 newborns indicated that the probability of events such as catheter obstruction occurring when the catheter tip is located in a peripheral region is up to seven times greater than when it is in a central position, with consequent impairment of blood return during aspiration^(24)^.

The “catheter insertion site” was identified by diagnosticians as a clinical indicator, but was not considered a predictor of the ND. A study evaluating 1,234 catheters that were placed in 918 hospitalized babies showed that poor positioning in the first 72 hours and over time was more common in upper limb catheters^(4)^.

In a study carried out in China, after the analysis of 40 patients undergoing catheterization, it was observed that, in comparison with the basilic, superficial temporal, or femoral veins, the PICC when accessed via the femoral vein can improve the success rate of catheterization, shorten insertion time and reduce complications, such as mechanical phlebitis, due to friction, because the blood vessels in the lower limbs are thicker and deeper^(25)^.

In this study, a high frequency of the clinical indicator “prematurity” was observed, data also found in other studies, where the use of PICC in newborns admitted to the ICU is more prevalent in premature neonates. The frequency of use of PICC in premature newborns is related to the predisposition to complications due to the immaturity of organs and systems, low weight, with a high risk of mortality, which make them require safe intravenous therapy^(26–31)^.

Prematurity (gestational age at birth<37 weeks) was associated with an increased risk of several complications, including malposition, as well as adverse events such as phlebitis, erythema, edema, and perfusion changes^(4)^.

These adverse events also represent a predisposing factor for the early removal of the catheter. A study conducted with 216 neonates using a PICC, most of whom were premature, indicated that early catheter removal occurred, on average, eight days after insertion. This removal was associated with maintenance difficulties, resulting in obstruction, breakage, phlebitis, and extrusion. Furthermore, the study pointed out that the use of multiple antibiotics, combined with parenteral nutrition plans, is linked to low neonatal weight, making it a relevant causal factor for early device removal^(27)^.

Prematurity was also identified as a risk factor for PICC removal due to suspected infection. The risk of infection is higher in neonates with greater clinical severity, requiring more frequent infusion therapy changes due to their instability, which leads to increased catheter manipulation^(28)^.

The clinical indicator “occurrence of adverse events” was identified as a predictor of the nursing diagnosis improper positioning of the peripherally inserted central catheter by the diagnostic evaluators. A randomized clinical trial observed an association between the final tip position of the PICC and the incidence of adverse events. In PICC procedures where the catheter tip was improperly positioned, the OR for adverse event occurrence was 4.10 times higher compared to procedures where the tip was centrally positioned^(21)^.

Thrombosis, embolism, vascular injuries, catheter obstruction, and displacement are potential complications associated with unplanned catheter removal. Among these, infections represent a significant concern, as direct access to the bloodstream and frequent manipulation increase the risk of bacterial colonization. Review studies have demonstrated an association between early catheter removal due to suspected infection and factors such as prematurity, low birth weight, non-central catheter tip positioning, and prolonged PICC use^(29)^.

A study revealed that the variables associated with the prevalence of complications during the use of PICC include male sex, low birth weight, age over 48 hours, more than three insertion attempts, procedural complications during catheter placement, frequent dressing changes, difficulty in catheter progression, and non-central positioning of the device. Therefore, the insertion and maintenance of PICCs by trained nurses is essential for the early identification of complication signs, contributing to the safe use of the device in neonates^(30)^.

The clinical indicator “newborn weight” was not considered a predictor for the ND improper positioning of the peripherally inserted central catheter by the diagnostic evaluators. However, it was observed that the lower the neonatal weight, the greater the likelihood of developing this diagnosis. In the institution where the research was conducted, the measurement protocol for catheter insertion considers a superficial anatomical measurement, which comprises the distance from the puncture site to the outer edge of the clavicle, reaching the third intercostal space.

A study aimed at developing diagrams and tables for recommending insertion depths of PICC in neonates, based on anthropometric parameters, demonstrated that the developed tables allowed for a rapid and precise determination of the recommended insertion depths for neonatal PICC^(6)^.

Studies exploring the relationship between neonatal anthropometric measurements and the appropriate depth for PICC insertion have shown a correlation between newborn weight and length, suggesting that these variables could serve as an anthropometric model for determining neonatal PICC insertion depth^(23,31)^.

It was observed that the modified measurement method for PICC resulted in a higher occurrence of central positioning and a lower incidence of intracardiac placement compared to the traditional method. In the group that used the traditional measurement, the frequency of central positioning was 2.3%, while intracardiac placement occurred in 72.7% of cases^(23)^.

The ND improper positioning of the peripherally inserted central catheter in neonatal units reinforces the importance of identifying measures to minimize catheter misplacement and adopting nursing interventions aimed at preventing the studied ND.

The findings of this study have significant implications for nursing clinical practice, contributing to diagnostic inference and the development of more precise interventions, favoring positive and sensitive outcomes in neonatal care.

The precise identification of clinical indicators plays a crucial role in preventing complications related to the use of PICC, promoting safety, reducing bloodstream infections, and minimizing neonatal discomfort. Furthermore, the findings reinforce the essential role of nurses in NICU, where evidence-based expertise enables the early detection of complications, the implementation of safe protocols, and the assurance of high-quality care for newborns.

Finally, conducting the study in a single institution represents a limitation, as it restricts the analysis to a specific institutional reality, reflecting localized practices, protocols, and care particularities that may impact the generalizability of the findings. Therefore, multicenter studies are recommended to expand the diversity of settings and enhance the external applicability of the generated evidence.

## CONCLUSION

The results of the present study confirmed the alternative hypothesis by demonstrating that the clinical indicators of the ND adverse location of the peripherally inserted central catheter presented significant accuracy measures. These findings demonstrate the reliability and accuracy of these indicators for diagnostic inference in neonates admitted to neonatal intensive care units. The clinical indicators “difficulty in catheter progression” and “absence of blood return” stood out due to their high specificity values, while “catheter insertion site,” “prematurity,” and “occurrence of adverse events” exhibited greater sensitivity, reinforcing the validity of the diagnostic inference based on these factors.

The study emphasizes the need for clinical validation of nursing diagnoses within the ICNP® framework in NICU, particularly concerning other statements within the terminological subset. Continuing this research could contribute to enhancing nursing taxonomies, strengthening clinical decision-making, and improving the quality of care for critically ill newborns.

Additionally, these findings may support evidence-based care protocols, reinforce standardized nursing language, and promote multicenter research, thereby expanding the applicability of results and their impact on neonatal patient safety.

## DATA AVAILABILITY

The data used during this study are available from the corresponding author upon reasonable request.
