# Supplementary material for: Clinical validation of the diagnosis adverse location of peripherally inserted central catheter in neonatology
Source: Rev Esc Enferm USP. 2025 Aug 4;59:e20250117. doi: 10.1590/1980-220X-REEUSP-2025-0117en (PMC12330225; doi:10.1590/1980-220X-REEUSP-2025-0117en)
Supplement: Translation

# Translation: Validação clínica do diagnóstico localização adversa do cateter central de inserção periférica em neonatologia

**DOI:** 10.1590/1980-220X-REEUSP-2025-0117pt

**Authors:** Nanete Caroline da Costa Prado, Richardson Augusto Rosendo da Silva, Harlon França de Menezes, Isabele Silva dos Santos, Rebecca Stefany da Costa Santos, Sérgio Danillo Santana de Lima Juraci, Dhyanine Morais de Lima Raimundo, Romanniny Hévillyn Silva Costa Almino

**Affiliations:** 1 Universidade Federal do Rio Grande do Norte, Programa de Pós-graduação em Enfermagem, Natal, RN, Brasil.

**Keywords:** Terminologia Padronizada em Enfermagem, Diagnóstico de Enfermagem, Estudo de Validação, Terapia Intensiva Neonatal, Cateterismo Venoso Central

## Abstract

**Objetivo:**

Determinar as medidas de acurácia dos indicadores clínicos do Diagnóstico de Enfermagem (DE) da Classificação Internacional da Prática de Enfermagem (CIPE^®^), localização adversa do cateter central de inserção periférica, em uma unidade de terapia intensiva neonatal (UTIN).

**Métodos:**

Estudo de acurácia diagnóstica realizado com 40 neonatos internados em uma UTIN no Nordeste brasileiro. A coleta de dados foi realizada por meio de exame clínico e aplicação de um instrumento institucional. A análise dos indicadores clínicos do respectivo DE foi realizada por meio das medidas de acurácia.

**Resultados:**

Dentre os participantes do estudo, a maioria era do sexo feminino e com baixo peso ao nascer. A prevalência do DE foi de 57,5%. Os indicadores clínicos dificuldade de progressão do cateter e ausência de retorno sanguíneo apresentaram valores elevados de especificidade. Já o local de inserção do cateter, a prematuridade e a ocorrência de eventos adversos apresentaram valores mais altos para sensibilidade.

**Conclusão:**

A inferência diagnóstica acurada possibilita a identificação precoce do posicionamento inadequado da ponta do cateter, oferecendo suporte ao enfermeiro na elaboração de intervenções precisas e eficazes para evitar complicações.

## INTRODUÇÃO

A Unidade de Terapia Intensiva Neonatal (UTIN) é um ambiente destinado ao atendimento de crianças de até 28 dias de vida, que oferece cuidados de alta complexidade e atendimento especializado em neonatologia. Os procedimentos são voltados à manutenção da vida, e comumente envolvem diversos procedimentos invasivos, a exemplo, o Cateter Central de Inserção Periférica (CCIP)^(1)^.

O termo CCIP refere-se a um cateter de longa permanência utilizado para a administração de soluções endovenosas. O dispositivo apresenta um formato delgado e flexível, permitindo sua condução por punção simples através de uma veia periférica sob visualização direta. A extremidade distal do cateter deve ser posicionada no terço distal da veia cava superior quando a punção for realizada a partir do membro superior, e na veia cava inferior quando a inserção ocorrer pelos membros inferiores^(2,3)^.

A inserção e o manejo do CCIP são competências do enfermeiro, conforme regulamentado pela Resolução nº 258/2001 do Conselho Federal de Enfermagem (COFEN). Essa norma estabelece diretrizes para a atuação do profissional de enfermagem na inserção do CCIP em neonatos, crianças e adultos, desde que capacitado e habilitado para realizar o procedimento^(4)^.

No contexto da UTIN, o enfermeiro exerce um papel essencial e qualificado no cuidado ao neonato em uso do cateter, exigindo habilidades clínicas específicas, conhecimento técnico atualizado e decisões seguras para minimizar riscos como infecções, migração da ponta do cateter, obstrução e trombose.

Entretanto, o uso do dispositivo frequentemente está associado ao mal posicionamento da extremidade distal durante a inserção, o que acarreta risco de condições adversas de diferentes graus de gravidade. Quando não identificada precocemente, essa complicação pode resultar em intervenções corretivas, que também apresentam riscos. Dessa forma, torna-se essencial que o profissional de saúde desenvolva habilidades avançadas de raciocínio clínico para reconhecer indicadores de localização adversa do cateter central de inserção periférica, minimizando sua ocorrência^(5,6)^.

Nesse cenário, o Processo de Enfermagem, pautado no pensamento crítico e julgamento clínico do enfermeiro, torna- se imprescindível para subsidiar o cuidado de maneira apropriada, promovendo a padronização da prática pela equipe de Enfermagem por meio de linguagem uniforme. Para tanto, a Classificação Internacional da Prática de Enfermagem (CIPE^®^) oferece o suporte necessário ao cuidado de Enfermagem ao possibilitar o desenvolvimento de subconjuntos terminológicos compostos pelos enunciados de diagnósticos, resultados e intervenções de enfermagem apropriados para áreas particulares de cuidado^(7,8)^.

Ademais, contribui para uma melhor implementação dos cuidados de enfermagem ao fornecer indicadores e segurança do paciente e para a realização de pesquisa, já que é uma terminologia de padrão internacional, com estrutura combinatória e enumerativa, reconhecida mundialmente, adaptável a diferentes ambientes, entre os quais o pediátrico e o neonatal.

Dessa forma, torna-se imprescindível o reconhecimento e a aplicação clínica de indicadores clínicos, por meio de estudos de validação, para garantir sua eficácia na prática e possibilitar a análise comparativa da replicação do modelo assistencial em diferentes contextos. Esse processo confere validade interna e externa ao estudo. Assim, a validação clínica consiste na coleta de dados que verificam a presença de indicadores clínicos associados aos enunciados, incluindo o Diagnóstico de Enfermagem (DE) de localização adversa do cateter central de inserção periférica.

A revisão do estado da arte sobre o objeto de estudo revelou a ausência de pesquisas específicas que avaliem indicadores clínicos para diagnósticos de enfermagem em recém-nascidos com CCIP, justificando a necessidade do presente estudo^(8,9)^. Os estudos de acurácia têm como objetivo identificar qual indicador melhor prediz a ocorrência do DE, orientando a escolha de intervenções mais adequadas para a assistência de enfermagem^(10)^. A realização desses estudos é essencial para garantir que a taxonomia de diagnósticos de enfermagem da CIPE® seja continuamente revisada e atualizada, gerando impacto na prática clínica do enfermeiro e promovendo maior segurança ao paciente.

Espera-se que este estudo auxilie enfermeiros que atuam em UTIN na identificação dos indicadores que melhor predizem a ocorrência do diagnóstico em questão. Dessa forma, contribuirá para uma inferência mais precisa, orientando a seleção e implementação de intervenções adequadas à assistência de enfermagem dessa população, reforçando a relevância da pesquisa.

Diante da problemática, levantaram-se os seguintes questionamentos: qual a prevalência do DE localização adversa do cateter central de inserção Periférica em neonatos internados em UTIN? Qual a acurácia dos seus indicadores clínicos?

Portanto, o objetivo do estudo em tela foi determinar as medidas de acurácia dos indicadores clínicos do DE da CIPE^®^ localização adversa do cateter central de inserção periférica em neonatos internados em UTIN.

## MÉTODO

### Desenho do Estudo

A presente pesquisa refere-se a um recorte de um projeto maior, onde o objetivo foi validar clinicamente os indicadores clínicos dos diagnósticos de enfermagem de um subconjunto terminológico da CIPE^®^ para recém-nascidos com CCIP em uma UTIN^(8,9)^. A partir da sua utilização na prática clínica, identificou-se a necessidade de atualização do mesmo e do mapeamento com a *Systematized Nomenclature of Medicine Clinical Terms* (SNOMED CT). Neste sentido, realizou-se uma nova validação de conteúdo por especialistas para posterior validação clínica por meio da acurácia diagnóstica.

Considerando o extenso número de diagnósticos presentes no referido subconjunto, optou-se, neste manuscrito, por apresentar apenas a acurácia do DE localização adversa do cateter central de inserção periférica, por ter sido o mais prevalente durante a validação clínica. Assim, trata-se de um estudo de acurácia diagnóstica, com delineamento analítico e abordagem quantitativa. O relatório foi elaborado seguindo as diretrizes da iniciativa *Standards for Reporting Studies of Diagnostic Accuracy* (STARD), garantindo clareza, transparência e qualidade na apresentação dos resultados^(11)^.

Foram mensurados os indicadores clínicos do referido DE, incluindo dificuldade de progressão do cateter, ausência de retorno sanguíneo, local de inserção do cateter, prematuridade e ocorrência de eventos adversos, conforme as seguintes definições operacionais:

- Dificuldade de progressão do cateter: caracterizada pela incapacidade de avançar o cateter por meio do introdutor ou da circulação periférica, pela impossibilidade de alcançar a distância previamente mensurada ou pela dificuldade de transpor o cateter da circulação periférica para a circulação central;
- Ausência de retorno sanguíneo: ocorre quando o insertor do cateter não obtém retorno do fluxo sanguíneo imediatamente após a inserção;
- Local de inserção do cateter: refere-se ao sítio escolhido pelo insertor para a introdução e progressão do cateter;
- Prematuridade: definida como o nascimento de um recém- nascido antes da 37ª semana completa de gestação, ou seja, com idade gestacional inferior a 37 semanas;
- Ocorrência de eventos adversos: complicações que podem surgir durante ou após a utilização do cateter, comprometendo a segurança e eficácia do tratamento intravenoso^(12,13)^.

### Local

O estudo foi realizado em uma UTIN no Nordeste brasileiro, vinculada ao Sistema Único de Saúde.

### População

A população do estudo foi composta por 40 neonatos internados na UTIN e em uso do CCIP em uma maternidade de cuidados críticos no Nordeste brasileiro. Além dos recém-nascidos, outros participantes compuseram o estudo, sendo quatro enfermeiros que atuaram como avaliadores dos estudos de casos.

### Critérios de Seleção

A seleção dos recém-nascidos deu-se por conveniência de forma consecutiva, adotando-se os seguintes critérios elegíveis de inclusão: recém-nascidos internados na UTIN em uso de CCIP e que permanecerem no setor até o término do tratamento. Os critérios de exclusão adotados foram: transferência do recém-nascido para outra instituição e recém-nascidos cujas mães eram adolescentes. A exclusão de mães adolescentes baseou-se nas normas éticas que regem pesquisas com seres humanos (Resolução CNS 466/12), uma vez que menores de idade, incluindo adolescentes, não possuem plena capacidade civil e, portanto, necessitam de um responsável legal para assinar o Termo de Consentimento Livre e Esclarecido (TCLE). Destaca-se que o TCLE foi lido, explicado e todas as dúvidas foram esclarecidas aos pais ou responsáveis pelo recém-nascido.

No que diz respeito à seleção dos enfermeiros, estes contavam com conhecimento e experiência em terapia intensiva neonatal e são insertores do cateter CCIP, bem como atenderam a todos os critérios de inclusão. Os critérios de inclusão foram: ter especialidade em neonatologia e experiência clínica assistencial de, pelo menos, cinco anos na área; ter curso de habilitação em inserção do cateter; ter experiência prática igual ou acima de dois anos em inserção do CCIP. Os profissionais afastados por licença ou férias durante o período de coleta de dados foram considerados critérios de exclusão.

O acesso aos enfermeiros especialistas ocorreu presencialmente no hospital onde a pesquisa foi realizada. Durante o contato inicial, foi feito o convite, e os objetivos do estudo, riscos e benefícios envolvidos foram explicitados. Posteriormente, aqueles que aceitaram participar assinaram o TCLE.

### Definição da Amostra

Para estabelecer a amostra dos recém-nascidos, utilizou- se a fórmula de cálculo de amostras para populações finitas, a fim de proporcionar de maneira imparcial, segura e científica a eficiência e eficácia desta pesquisa. O tamanho da amostra foi calculado seguindo o seguinte procedimento: foi atribuído um nível de significância (a) de 5% e uma margem de erro de 0,05. Quando a proporção era desconhecida, adotou-se p = 0,5, considerando a variância máxima. Para a seleção dos especialistas do estudo, utilizou-se amostragem não probabilística intencional.

### Coleta de Dados

Para o alcance do objetivo, o projeto global foi operacionalizado de acordo as seguintes fases: 1) Implementação do Processo de Enfermagem de acordo com as etapas: avaliação de enfermagem, DE, planejamento de enfermagem, implementação e evolução de enfermagem; 2) Elaboração dos estudos de casos e enunciados dos diagnósticos/resultados e intervenções de enfermagem (DE/RE/IE); 3) Análise de concordância entre os enfermeiros especialistas; 4) Acurácia dos indicadores clínicos dos diagnósticos de enfermagem do subconjunto terminológico^(8)^. O período de coleta de dados foi de outubro a dezembro de 2024.

Os enunciados dos DE e seus respectivos indicadores clínicos foram testados na população de neonatos, utilizando as seguintes medidas de acurácia: sensibilidade, especificidade, valor preditivo positivo e negativo, razão de verossimilhança positiva e negativa.

Os dados dos pacientes foram coletados por meio de exame físico e da análise de registros de enfermagem contidos nos prontuários da amostra. A coleta de dados e a inferência diagnóstica foram realizadas pela pesquisadora principal, em conjunto com uma aluna do curso de doutorado em Enfermagem.

Para o estudo, utilizou-se um instrumento baseado no modelo teórico de Betty Neuman, estruturado nas seguintes seções: dados sociodemográficos, avaliação, diagnósticos, planejamento, implementação e evolução de enfermagem. Antes da aplicação definitiva, foi realizado um teste-piloto, previamente aprovado pelo comitê de ética em pesquisa, com o objetivo de verificar a adequação do instrumento. Ressalta-se que os participantes do teste-piloto não foram incluídos no estudo principal.

Os especialistas receberam planilhas desenvolvidas pela dupla, cada uma correspondente a um paciente e contendo todos os termos e DE/RE e IE de acordo com o modelo teórico empregado. As planilhas também incluíam informações complementares sobre dados socioeconômicos, clínicos e observações relevantes ao processo de inferência diagnóstica. Cada um avaliou separadamente a presença ou ausência dos DE, RE e IE em cada uma das planilhas recebidas.

Para esta pesquisa, foram considerados clinicamente validados os DE/RE e IE identificados em recém-nascidos, descritos nos estudos de caso e incluídos no subconjunto terminológico. A análise de concordância entre os enfermeiros juízes foi realizada por meio do coeficiente de concordância de Kappa (k), sendo que os especialistas analisaram os estudos de caso e os enunciados listados.

A última etapa do estudo consistiu na avaliação da acurácia dos indicadores clínicos dos DE do subconjunto terminológico, com o objetivo de identificar a exatidão desses indicadores na população de neonatos em uso de CCIP. Assim, analisou-se qual indicador apresentava maior precisão na predição da ocorrência dos DE investigados.

No presente estudo, os indicadores clínicos foram definidos com base na literatura, que subsidia a tomada de decisão clínica. Considerando a limitação do número de páginas da revista, este artigo se concentrará exclusivamente na acurácia dos indicadores clínicos do DE da CIPE “localização adversa do cateter central de inserção periférica”, por ter sido o mais prevalente na pesquisa. Essa abordagem se faz necessária devido à extensão do número de indicadores dos DE que compõem o subconjunto. Destaca-se que outros resultados do projeto global serão publicados em futuras produções científicas.

### Análise e Tratamento dos Dados

A análise dos dados deu-se pela verificação do nível de concordância e nível de consistência (fidedignidade) da opinião dos juízes (enfermeiros especialistas) quanto aos demais itens do subconjunto terminológico, sendo aplicado o coeficiente de concordância de Kappa (k)^(14)^.

Este índice foi aplicado também na verificação da concordância inter-avaliadores em relação a cada item. O coeficiente de concordância de Kappa avalia a proporção de concordância que varia de “menos 1” a “mais 1”; quanto mais próximo de 1, melhor o nível de concordância entre os observadores. Cada juiz indicou se o item era relevante e se as respectivas definições eram claras e precisas, mediante escolha de uma opção dentre as cinco respostas sugeridas (concordo totalmente – 1; concordo parcialmente – 2; indiferente – 3; discordo parcialmente – 4; discordo totalmente – 5).

Para cada opção foi atribuída uma pontuação: concordo totalmente – 1,0; concordo parcialmente – 0,75; indiferente – 0,50; discordo parcialmente – 0,25; discordo totalmente – 0,0. Este critério foi utilizado tanto para a pertinência quanto para a aplicabilidade de cada item. Como critério de aceitação, foi estabelecida uma concordância ≥ 0,80 entre os juízes, sendo considerado um nível bom.

A concordância entre os quatro enfermeiros especialistas foi avaliada por meio do Coeficiente Kappa (k), classificada da seguinte forma: pobre (de 0 a 0,19); relativa (de 0,20 a 0,39); moderada (de 0,40 a 0,59); substancial (de 0,60 a 0,79); quase perfeita (de 0,80 a 0,99); e perfeita (igual a 1)^(15)^.

O teste não paramétrico de Kruskal-Wallis foi utilizado para verificar se houve diferenças nas médias das avaliações dos enfermeiros especialistas. O nível de significância adotado foi de p ≤ 0,05; assim, uma diferença entre as médias foi considerada estatisticamente significativa quando o valor de p era menor ou igual a esse limite. Para avaliar a confiabilidade e aplicabilidade do subconjunto em diferentes contextos, utilizou- se o Coeficiente de Correlação Intraclasse (CCI), onde CCI ≥ 0,75 indica excelente confiabilidade; CCI entre 0,40 e 0,75 denota confiabilidade satisfatória; e CCI < 0,40 é considerado pobre^(14,15)^.

Os dados referentes ao DE e RE foram analisados por meio de estatística descritiva, considerando frequências absoluta e relativa. As informações sobre IE foram apresentadas de forma agrupada, seguindo as categorias do modelo teórico, e discutidas conforme suas frequências. Posteriormente, foram organizados em uma planilha eletrônica, e, após dupla conferência de digitação, foram exportados para o *Statistical Package for Social Science* (SPSS), versão 20.0 Windows. Após codificação e tabulação, os dados foram analisados por meio de leitura reflexiva e estatística, com aplicação do teste de Kappa utilizando o Online Kappa Calculator.

Com base nos objetivos do estudo e em sua natureza, foram formuladas as seguintes hipóteses: Hipótese Nula (H_0_): Os indicadores clínicos do DE localização adversa do cateter central de inserção periférica não apresentam medidas de acurácia significativas para a inferência diagnóstica em neonatos internados em UTIN; Hipótese Alternativa (H_1_): Os indicadores clínicos do DE localização adversa do cateter central de inserção periférica apresentam medidas de acurácia significativas para a inferência diagnóstica em neonatos internados em UTIN.

A análise da validação clínica foi conduzida em duas fases. A primeira fase envolveu a caracterização da população, utilizando estatística descritiva. Na segunda fase, foram examinadas as medidas de acurácia dos indicadores clínicos em relação ao DE. Essas medidas foram calculadas com base na inferência diagnóstica da presença do DE, estabelecendo-se um ponto de corte de 50%. Para avaliar as associações entre as variáveis qualitativas e os indicadores clínicos, aplicou-se o Teste Qui-Quadrado ou o Teste Exato de Fischer.

### Aspectos Éticos

O estudo foi aprovado em Comitê de Ética em Pesquisa com o parecer número 7.171.001 de 2024 e obedeceu aos princípios éticos da Resolução nº 466, de 12 de dezembro de 2012, do Conselho Nacional de Saúde, que discorre sobre a pesquisa envolvendo seres humanos.

## RESULTADOS

Participaram deste estudo 40 recém-nascidos, dos quais 65% (26) eram do sexo feminino com predominância de bebês nascidos de parto cesárea (70%), baixo peso ao nascer (65%) e com média de APGAR de 6,46 e 8,52 no primeiro e quinto minuto de vida, respectivamente. O tempo médio de permanência do CCIP foi de 10,23 dias.

As principais indicações para a inserção do cateter foram antibioticoterapia (80%) e nutrição parenteral (65%). Quanto ao diagnóstico clínico, as afecções respiratórias (75%) e a prematuridade (70%) foram os mais prevalentes. O tipo de cateter utilizado foi o de único lúmen e de silicone. No tocante aos sítios de inserção, os membros superiores foram os mais prevalentes (55%). Todos os cateteres foram inseridos por enfermeiros.

Em relação a prevalência dos indicadores clínicos do DE da CIPE^®^, localização adversa do cateter central de inserção periférica, observou-se: “dificuldade de progressão do cateter” (57,5%), “ausência de retorno sanguíneo” (55%), “local de inserção do cateter (membros superiores)” (55%), “ocorrência de eventos adversos” (50%), “prematuridade (< 37 semanas)” (47,5%), “peso do recém-nascido” (45%), “presença do cateter venoso umbilical” (32,5%), “presença de outro cateter de inserção periférica” (27,5%), “malformação congênita” (7,5%).

A Tabela 1 apresenta as razões de prevalência dos indicadores clínicos analisados segundo a ocorrência do DE localização adversa do cateter central de inserção periférica em neonatos internados em UTIN. Os dados evidenciam os indicadores clínicos que apresentaram associação estatisticamente significativa com o diagnóstico, assim como suas razões de prevalência e intervalos de confiança. Esses achados destacam os fatores mais relevantes para a inferência diagnóstica, contribuindo para uma análise mais precisa e fundamentada.

**Tabela 1:** Razões de prevalência dos indicadores clínicos segundo a ocorrência do DE localização adversa do cateter central de inserção periférica em recém-nascidos em UTIN – Natal, RN, Brasil, 2024.

Variáveis	DE localização adversa do cateter central de inserção periférica						
Indicadores clínicos	Presente		Ausente		Total		Estatísticas
	N	%	N	%	N	%	
Dificuldade de progressão do cateter							
Presente	23,0	57,5	0,0	0,0	23,0	57,5	*p* = 0,001*
Ausente	0,0	0,0	17,0	42,5	17,0	42,5	RP^†^ = 4,98^†^
Total	23,0	57,5	17,0	42,5	40,0	100,0	IC^‡^95%: 3,09–5,88^‡^
Ausência de retorno sanguíneo							
Presente	21,0	52,5	1,0	2,5	22,0	55,0	*p* = 0,001*
Ausente	4,0	10,0	14,0	35,0	18,0	45,0	RP^†^ = 4,02^†^
Total	23,0	57,5	17,0	42,5	40,0	100,0	IC^‡^95%: 2,71–5,17^‡^
Local de inserção do cateter (membros superiores)							
Presente	19,0	47,5	3,0	7,5	22,0	55,0	*p* = 0,038*
Ausente	4,0	10,0	14,0	35,0	18,0	45,0	RP^†^ = 3,95^†^
Total	23,0	57,5	17,0	42,5	40,0	100,0	IC^‡^95%: 2,15–4,80^‡^
Ocorrência de eventos adversos							
Presente	18,0	45,0	2,0	5,0	20,0	50,0	*p* = 0,002*
Ausente	5,0	12,5	15,0	37,5	20,0	50,0	RP^†^ = 2,98^†^
Total	23,00	57,5	17,0	42,5	40,0	100,0	IC^‡^95%: 1,84–3,56^‡^
Prematuridade (< 37 semanas)							
Presente	16,0	40,0	3,0	7,5	19,0	47,5	*p* = 0,012*
Ausente	7,0	17,5	14,0	35,0	21,0	52,5	RP^†^ = 2,92^†^
Total	23,0	57,5	17,0	42,5	40,0	100,0	IC^‡^95%: 1,69–3,28^‡^
Peso do recém-nascido							
Presente	11,0	27,5	7,0	17,5	18,0	45,0	*p* = 0,332§
Ausente	12,0	30,0	10,0	25,0	22,0	55,0	RP^†^ = 1,48^†^
Total	23,0	57,5	17,0	42,5	40,0	100,0	IC^‡^95%: 0,86–1,98^‡^
Presença do Cateter Venoso Umbilical							
Presente	9,0	22,5	4,0	10,0	13,0	32,5	*p* = 0,217*
Ausente	14,0	35,0	13,0	32,5	27,0	67,5	RP^†^ = 1,46^†^
Total	23,0	57,5	17,0	42,5	40,0	100,0	IC^‡^95%: 0,71–1,92^‡^
Presença de outro Cateter Venoso de Inserção Periférica							
Presente	8,0	20,0	3,0	7,5	11,0	27,5	*p* = 0,402*
Ausente	15,0	37,5	14,0	35,0	29,0	72,5	RP^†^ = 1,34^†^
Total	23,0	57,5	17,0	42,5	40,0	100,0	IC^‡^95%: 0,97–2,28^‡^
Malformação congênita							
Presente	2,0	5,0	1,0	2,5	3,0	7,5	*p* = 0,619*
Ausente	21,0	52,5	16,0	40,0	37,0	92,5	RP^†^ = 1,21^†^
Total	23,0	57,5	17,0	42,5	40,0	100,0	IC^‡^95%: 0,82–2,12^‡^

*teste exato de Fisher;

^†^RP = razão de prevalência;

^‡^IC = intervalo de confiança de 95%;

^§^teste de qui-quadrado de Pearson; p < 0,05.

Com base na Tabela 1, os indicadores clínicos “Dificuldade de progressão do cateter”, “Ausência de retorno sanguíneo”, “Local de inserção do cateter (membros superiores)”, “Ocorrência de eventos adversos” e “Prematuridade (< 37 semanas)” apresentaram relação de risco com o diagnóstico de enfermagem (DE) estudado. Ademais, observou-se um aumento na probabilidade dos RNs desenvolverem o DE localização adversa do cateter central de inserção periférica, conforme as seguintes razões de prevalência (RP): 5 vezes (RP = 4,98), 4 vezes (RP = 4,02), 4 vezes (RP = 3,95), 3 vezes (RP = 2,98),3 vezes (RP = 2,92), respectivamente.

Por outro lado, os indicadores “Peso do recém-nascido”, “Presença do Cateter Venoso Umbilical”, “Presença de outro Cateter de Inserção Periférica” e “Malformação congênita” não apresentaram associação estatisticamente significativa com o DE localização adversa do cateter central de inserção periférica.

A Tabela 2 detalha as medidas de acurácia dos indicadores clínicos relacionados ao DE localização adversa do cateter central de inserção periférica em recém-nascidos internados em Unidade de Terapia Intensiva Neonatal.

**Tabela 2:** Medidas de acurácia dos indicadores clínicos do DE, localização adversa do cateter central de inserção periférica em recém-nascidos em Unidade de Terapia Intensiva Neonatal – Natal, RN, Brasil, 2024.

Indicadores clínicos	Se*	Es^†^	VPP^‡^	VPN^§^	RVP^||^ (IC 95%)	RVN^¶^ (IC 95%)	ORD** (IC 95%)
Dificuldade de progressão do cateter	100,0	45,28	100,0	16,97	9,00 (7,87–11,67)	0,67 (0,31–0,91)	8,92 (6,91–10,68)
Ausência de retorno sanguíneo	98,34	51,10	98,41	29,55	6,31 (4,24–8,12)	0,62 (0,25–0,87)	6,21 (4,16–8,74)
Local de inserção do cateter (membros superiores)	57,31	92,30	97,12	23,45	4,84 (2,14–6,92)	0,53 (0,41–0,77)	5,00 (3,24–7,61)
Ocorrência de eventos adversos	48,87	89,93	91,39	19,38	3,33 (1,18–5,73)	0,30 (0,19–0,64)	4,93 (2,84–6,75)
Prematuridade (< 37 semanas)	54,21	94,85	99,82	22,31	3,46 (1,28–5,98)	0,76 (0,62–0,83)	6,90 (4,55–8,65)
Peso do recém-nascido	13,26	54,58	97,50	28,44	1,26 (0,02–2,63)	0,88 (0,53–1,69)	2,76 (0,95–4,08)
Presença do Cateter Venoso Umbilical	31,16	64,07	57,41	26,84	1,46 (0,65–3,86)	0,65 (0,21–1,10)	1,93 (0,89–4,32)
Presença de outro Cateter Venoso de Inserção Periférica	33,08	64,13	96,30	22,17	1,98 (0,85–4,12)	0,82 (0,55–1,17)	2,11 (0,91–4,85)
Malformação congênita	3,29	44,55	97,50	18,42	1,37 (0,14–3,29)	0,55 (0,13–1,12)	1,41 (0,67–3,28)

*Se = sensibilidade;

^†^Es = especificidade;

^‡^VPP = valor preditivo positivo;

^§^VPN = valor preditivo negativo;

^||^RVP = razão de verossimilhança positiva;

^¶^RVN = razão de verossimilhança negativa;

**ORD = *Odds Ratio* diagnóstica.

A prevalência do DE localização adversa do cateter central de inserção periférica foi de 57,5%. Os indicadores clínicos “dificuldade de progressão do cateter” e “ausência de retorno sanguíneo” apresentaram valores elevados de especificidade, além de valores de verossimilhança e ORD estatisticamente significativos, sugerindo serem bons indicadores para confirmar a presença do diagnóstico.

Além disso, os indicadores clínicos “local de inserção do cateter”, “prematuridade” e “ocorrência de eventos adversos” apresentaram valores mais altos de sensibilidade, acompanhados de valores de verossimilhança e ORD estatisticamente significativos, indicando que esses indicadores possuem as melhores medidas de acurácia para a inferência do DE estudado.

## DISCUSSÃO

O DE localização adversa do cateter central de inserção periférica foi validado clinicamente pelas medidas de acurácia, corroborando a literatura, que enfatiza a importância da vigilância quanto ao posicionamento da ponta do cateter, pois a incidência de mau posicionamento varia entre 10% e 60%. Tal inadequação pode resultar em eventos adversos significativos, como tamponamento cardíaco, derrame pleural e pericárdico, infiltração, lesão endotelial, entre outros, representando um risco considerável para a segurança do paciente^(16–18)^.

Considera-se que a ocorrência de posicionamento inadequado do cateter CCIP em recém-nascidos sob cuidados intensivos é significativamente mais comum, estimada em cerca de três vezes maior em comparação com outros acessos centrais^(19)^.

Foi relatado que a migração do CCIP ocorre nas primeiras 24-72 horas após a inserção^(12,20)^. Portanto, alguns pesquisadores sugeriram a realização de uma repetição da radiografia 24 horas após a inserção de um CCIP para identificar a migração, enquanto outros sugeriram a realização de vigilância por imagem até 72 horas após a inserção do CCIP^(12)^.

Sobre os fatores de risco, o indicador clínico “dificuldade de progressão do cateter” foi considerado preditor do DE em tela. Tal achado pode ser evidenciado na medida em que a progressão inadequada ocorre devido ao trajeto incorreto do CCIP, resultando no enovelamento do cateter e avanço em direção aos vasos colaterais próximos à circulação central (veias subclávias, jugulares e axilares), sendo associada ao desfecho de posição periférica^(21)^. A dificuldade de progressão do CCIP pode ser definida como incapacidade de passar o cateter pelo introdutor ou pela circulação periférica, incapacidade de passar o cateter até a distância previamente mensurada ou incapacidade de passar o cateter da circulação periférica para a circulação central^(12)^.

Em um estudo realizado em UTIN, foi observada uma dificuldade na progressão em 63 procedimentos (25,8%), indicando uma correlação significativa com a posição inicial da ponta do cateter. A dificuldade de progressão foi relatada em 50% dos cateteres com posição inicial não central e em 22% dos cateteres com posição central^(22)^. Outro estudo que corrobora os achados da presente pesquisa foi realizado com 141 neonatos, o qual identificou que a dificuldade de progressão do CCIP durante a inserção esteve associada à migração da ponta do cateter (p < 0,01)^(12)^.

A “ausência de retorno sanguíneo” logo após a inserção do cateter, também chamado de teste de fluxo e refluxo foi considerada fator de risco para o falso trajeto do CCIP e foi um indicador clínico relacionado ao DE estudado, sendo também considerado um preditor para a localização adversa do cateter central de inserção periférica em neonatologia. Tal achado corrobora a literatura, a qual destaca que há muito, além da dificuldade na progressão do cateter durante a inserção, a aspiração sanguínea prejudicada é relatada como fator preditor de que pode ter ocorrido mau implante do cateter^(23)^.

Uma pesquisa que envolveu 100 recém-nascidos indicou que a probabilidade de eventos como a obstrução do cateter ocorrer quando a ponta do cateter está localizada em região periférica é até sete vezes maior do que quando está na posição central, com consequente prejuízo no retorno sanguíneo durante a aspiração^(24)^.

O “local de inserção do cateter” foi identificado pelos diagnosticadores como indicador clínico, mas não foi considerado preditor do DE. Estudo que avaliou 1.234 cateteres que foram colocados em 918 bebês hospitalizados demonstrou que o mau posicionamento nas primeiras 72 horas e ao longo do tempo foi mais comum em cateteres de membros superiores^(4)^.

De acordo com estudo realizado na China, em comparação com as veias basílicas, temporal superficial ou femoral, observou- se após análise de 40 pacientes submetidos ao cateterismo, que o CCIP quando acessado por veia femoral pode melhorar a taxa de sucesso do cateterismo, encurtar o tempo de inserção e reduzir as complicações, como a flebite mecânica, devido a fricção, pelo fato de os vasos sanguíneos dos membros inferiores serem mais espessos e profundos^(25)^.

Observou-se, neste estudo, a alta frequência do indicador clínico “prematuridade”, dado também encontrado em outros estudos, onde o uso de CCIP em recém-nascidos internados na UTI apresenta maior prevalência em neonatos prematuros. A frequência do uso do CCIP em recém-nascidos prematuros está relacionada à predisposição a complicações devido à imaturidade de órgãos e sistemas, baixo peso, com alto risco de mortalidade, que os fazem necessitar de terapia endovenosa segura^(26)^.

A prematuridade (idade gestacional ao nascer<37 semanas) foi associada a um risco aumentado de várias complicações, incluindo mau posicionamento, além de eventos adversos como flebite, eritema, edema e alterações de perfusão^(4)^.

Esses eventos adversos também representam um fator predisponente para a remoção precoce do cateter. Um estudo realizado com 216 neonatos em uso de CCIP, sendo a maioria prematuros, apontou que a retirada precoce do cateter ocorreu, em média, 8 dias após a inserção. Essa remoção foi associada à dificuldade de manutenção, resultando em obstrução, quebra, flebite e extrusão. Além disso, o estudo indicou que o uso de múltiplos antibióticos, em conjunto com o plano de nutrição parenteral, está associado ao baixo peso da população neonatal, sendo um fator causal relevante para a remoção antecipada do dispositivo^(27)^.

A prematuridade também se mostrou um fator de risco para a remoção do CCIP por suspeita de infecção. O risco de infecção é mais frequente em neonatos com maior gravidade clínica, o que exige trocas mais frequentes da terapia infusional devido à sua instabilidade, resultando em uma maior manipulação do cateter^(28)^.

O indicador clínico “ocorrência de eventos adversos” foi identificado como preditivo do DE localização adversa do cateter central de inserção periférica pelos diagnosticadores. Um ensaio clínico randomizado observou uma associação entre a localização final da ponta do CCIP e a incidência de eventos adversos. Nos procedimentos de CCIP em que a ponta estava mal posicionada, verificou-se que o ODR para a ocorrência de eventos adversos foi 4,10 vezes maior em comparação aos procedimentos em que a ponta estava centralmente posicionada^(21)^.

A trombose, embolia, lesões vasculares, obstrução do cateter e deslocamento são complicações potenciais associadas à remoção não planejada dos cateteres. Dentre essas, as infecções representam uma preocupação significativa, pois o acesso direto à corrente sanguínea e a manipulação frequente aumentam o risco de colonização bacteriana. Estudos de revisão apontam a associação entre a retirada precoce do cateter por suspeita de infecção e fatores como prematuridade, baixo peso, posicionamento não central da ponta do cateter e uso prolongado do CCIP^(29)^.

Um estudo revelou que as variáveis associadas à prevalência de complicações durante o uso do CCIP incluem: sexo masculino, baixo peso, idade superior a 48 horas de vida, mais de três tentativas de inserção do CCIP, intercorrências durante a inserção, trocas frequentes de curativo, dificuldade de progressão do cateter e localização não central do dispositivo. Portanto, a inserção e manutenção do CCIP por enfermeiros capacitados é essencial para a identificação precoce de sinais de complicações, contribuindo para a segurança no uso do dispositivo em neonatos^(30)^.

O indicador clínico “peso do recém-nascido” não foi considerado preditor do DE localização adversa do cateter central de inserção periférica pelos diagnosticadores. No entanto, observou-se que quanto menor o peso do neonato, maior a chance da ocorrência desse diagnóstico. Na instituição onde a pesquisa foi realizada, o protocolo de mensuração para a inserção do cateter considera a medida anatômica superficial, que compreende a distância do local de punção até a extremidade externa da clavícula, chegando ao 3º espaço intercostal.

Um estudo que teve como objetivo desenvolver diagramas e tabelas para a recomendação de profundidades de inserção do CCIP em neonatos, com base em parâmetros antropométricos, evidenciou que as tabelas desenvolvidas permitiram uma determinação rápida e precisa das profundidades de inserção recomendadas do CCIP em neonato^(6)^.

Estudos que exploraram a relação entre as medidas antropométricas neonatais e a profundidade apropriada de inserção do CCIP demonstraram uma correlação entre o peso e o comprimento do recém-nascido, sugerindo que essas variáveis podem servir como modelo antropométrico para a determinação da profundidade de inserção do CCIP neonatal^(23,31)^.

Observou-se que o método de mensuração modificado do CCIP resultou em uma maior ocorrência de localização central e menor incidência de localização intracardíaca, quando comparado ao método tradicional. No grupo que utilizou a medida tradicional, a frequência de posicionamento central foi de 2,3%, enquanto a localização intracardíaca ocorreu em 72,7% dos casos^(23)^.

O DE localização adversa do cateter venoso central de inserção periférica em unidades neonatais reforça a importância da identificação de medidas que minimizem falhas na localização do cateter e da adoção de intervenções de enfermagem voltadas para a prevenção do DE estudado.

Os achados deste estudo apresentam implicações significativas para a prática clínica de enfermagem, contribuindo para a inferência diagnóstica e a formulação de intervenções mais precisas, favorecendo desfechos positivos e sensíveis na assistência neonatal.

A identificação precisa dos indicadores clínicos auxilia na prevenção de complicações relacionadas ao uso do CCIP, promovendo segurança, redução de infecções da corrente sanguínea e minimização do desconforto neonatal. Além disso, os achados reforçam o papel essencial do enfermeiro na UTIN, cuja atuação qualificada e baseada em evidências possibilita a identificação precoce de complicações, a implementação de protocolos seguros e a garantia da qualidade do cuidado ao recém-nascido.

Por fim, a realização do estudo em um único serviço constitui uma limitação, pois restringe a análise a uma única realidade institucional, refletindo práticas, protocolos e especificidades assistenciais que podem comprometer a generalização dos achados. Assim, recomenda-se a realização de estudos multicêntricos, que ampliem a diversidade de cenários e possibilitem a aplicabilidade externa das evidências produzidas.

## CONCLUSÃO

Os resultados do presente estudo confirmaram a hipótese alternativa ao demonstrarem que os indicadores clínicos do DE localização adversa do cateter central de inserção periférica apresentaram medidas de acurácia significativas. Esses achados evidenciam a confiabilidade e a precisão desses indicadores para a inferência diagnóstica em neonatos internados em unidades de terapia intensiva neonatal. Os indicadores clínicos “dificuldade de progressão do cateter” e “ausência de retorno sanguíneo” destacaram-se pelos elevados valores de especificidade, enquanto “local de inserção do cateter”, “prematuridade” e “ocorrência de eventos adversos” apresentaram maior sensibilidade, reforçando a validade da inferência diagnóstica baseada nesses fatores.

O estudo enfatiza a necessidade de validação clínica dos diagnósticos de enfermagem da CIPE® na UTIN, especialmente em relação aos demais enunciados do subconjunto terminológico. A continuidade dessa investigação pode contribuir para o aprimoramento das taxonomias de enfermagem, fortalecendo a tomada de decisão clínica e qualificando a assistência ao recém-nascido crítico.

Além disso, os achados poderão embasar protocolos assistenciais baseados em evidências, fortalecer a linguagem padronizada na enfermagem e fomentar pesquisas multicêntricas, ampliando a aplicabilidade dos resultados e seu impacto na segurança do paciente neonatal.

## DISPONIBILIDADE DE DADOS

Os dados utilizados durante este estudo estão disponíveis com o autor correspondente mediante solicitação razoável.

# Translation: Validación clínica del diagnóstico de localización adversa del catéter central de inserción periférica en neonatología

**DOI:** 10.1590/1980-220X-REEUSP-2025-0117es

**Authors:** Nanete Caroline da Costa Prado, Richardson Augusto Rosendo da Silva, Harlon França de Menezes, Isabele Silva dos Santos, Rebecca Stefany da Costa Santos, Sérgio Danillo Santana de Lima Juraci, Dhyanine Morais de Lima Raimundo, Romanniny Hévillyn Silva Costa Almino

**Affiliations:** 1 Universidade Federal do Rio Grande do Norte, Programa de Pós-graduação em Enfermagem, Natal, RN, Brasil.

**Keywords:** Terminología Normalizada de Enfermería, Diagnóstico de Enfermería, Estudio de Validación, Cuidado Intensivo Neonatal, Cateterismo Venoso Central

## Abstract

**Objetivo:**

Determinar las medidas de precisión de los indicadores clínicos de Diagnóstico de Enfermería (DE) de la Clasificación Internacional de la Práctica de Enfermería (CIPE^®^), Ubicación adversa de un catéter central de inserción periférica en una unidad de cuidados intensivos neonatales (UCIN).

**Métodos:**

Estudio de precisión diagnóstica realizado con 40 neonatos internados en una UCIN del Nordeste de Brasil. La recolección de datos se realizó mediante examen clínico y aplicación de un instrumento institucional. El análisis de los indicadores clínicos del respectivo DE se realizó mediante medidas de precisión.

**Resultados:**

Entre los participantes del estudio, la mayoría eran mujeres y tenían bajo peso al nacer. La prevalencia de DE fue del 57,5%. Los indicadores clínicos dificultad en la progresión del catéter y ausencia de retorno sanguíneo mostraron valores elevados de especificidad. El sitio de inserción del catéter, la prematuridad y la ocurrencia de eventos adversos presentaron valores de sensibilidad mayores.

**Conclusión:**

La inferencia diagnóstica precisa permite la identificación temprana de una posición inadecuada de la punta del catéter, lo que ayuda a los enfermeros a desarrollar intervenciones precisas y efectivas para evitar complicaciones.

## INTRODUCCIÓN

La Unidad de Terapia Intensiva Neonatal (UTIN) es un entorno destinado a la atención de niños de hasta 28 días de vida, que ofrece cuidados de alta complejidad y atención especializada en neonatología. Los procedimientos están orientados al mantenimiento de la vida y suelen implicar diversas intervenciones invasivas, como el catéter central de inserción periférica (CCIP)^(1)^.

El término CCIP se refiere a un catéter de larga permanencia utilizado para la administración de soluciones intravenosas. El dispositivo tiene una forma delgada y flexible, lo que permite su inserción mediante una simple punción a través de una vena periférica bajo visualización directa. El extremo distal del catéter debe colocarse en el tercio distal de la vena cava superior cuando la punción se realiza desde el miembro superior, y en la vena cava inferior cuando la inserción se realiza desde los miembros inferiores^(2,3)^.

La inserción y el manejo del CCIP son competencias del enfermero, según lo regulado por la Resolución n.º 258/2001 del Consejo Federal de Enfermería (COFEN). Esta norma establece directrices para la actuación del profesional de enfermería en la inserción del CCIP en neonatos, niños y adultos, siempre que esté capacitado y habilitado para realizar el procedimiento^(4)^.

En el contexto de la UCIN, el enfermero desempeña un papel esencial y cualificado en el cuidado del recién nacido que utiliza el catéter, lo que requiere habilidades clínicas específicas, conocimientos técnicos actualizados y decisiones seguras para minimizar riesgos como infecciones, migración de la punta del catéter, obstrucción y trombosis.

Sin embargo, el uso del dispositivo se asocia frecuentemente con una mala colocación del extremo distal durante la inserción, lo que conlleva el riesgo de condiciones adversas de diferente gravedad. Cuando no se identifica precozmente, esta complicación puede dar lugar a intervenciones correctivas, que también presentan riesgos. Por lo tanto, es esencial que el profesional de la salud desarrolle habilidades avanzadas de razonamiento clínico para reconocer los indicadores de ubicación adversa del catéter central de inserción periférica, minimizando su ocurrencia^(5,6)^.

En este contexto, el proceso de enfermería, basado en el pensamiento crítico y el juicio clínico del enfermero, se vuelve imprescindible para respaldar la atención de manera adecuada, promoviendo la estandarización de la práctica por parte del equipo de enfermería mediante un lenguaje uniforme. Para ello, la Clasificación Internacional de la Práctica de Enfermería (CIPE^®^) ofrece el apoyo necesario a la atención de enfermería al permitir el desarrollo de subconjuntos terminológicos compuestos por enunciados de diagnósticos, resultados e intervenciones de enfermería apropiados para áreas particulares de la atención^(7,8)^.

Además, contribuye a una mejor implementación de los cuidados de enfermería al proporcionar indicadores y seguridad al paciente y a la realización de investigaciones, ya que es una terminología de estándar internacional, con estructura combinatoria y enumerativa, reconocida mundialmente, adaptable a diferentes entornos, entre ellos el pediátrico y el neonatal.

Por lo tanto, es imprescindible el reconocimiento y la aplicación clínica de los indicadores clínicos, mediante estudios de validación, para garantizar su eficacia en la práctica y permitir el análisis comparativo de la réplica del modelo asistencial en diferentes contextos. Este proceso confiere validez interna y externa al estudio. Así, la validación clínica consiste en la recolección de datos que verifican la presencia de indicadores clínicos asociados a los enunciados, incluyendo el Diagnóstico de Enfermería (DE) de localización adversa del catéter central de inserción periférica.

La revisión del estado del arte sobre el objeto de estudio reveló la ausencia de investigaciones específicas que evalúen los indicadores clínicos para los diagnósticos de enfermería en recién nacidos con CCIP, lo que justifica la necesidad del presente estudio^(8,9)^. Los estudios de precisión tienen como objetivo identificar qué indicador predice mejor la aparición del DE, orientando la elección de las intervenciones más adecuadas para la asistencia de enfermería^(10)^. La realización de estos estudios es esencial para garantizar que la taxonomía de diagnósticos de enfermería de la CIPE® se revise y actualice continuamente, generando un impacto en la práctica clínica del enfermero y promoviendo una mayor seguridad para el paciente.

Se espera que este estudio ayude a los enfermeros que trabajan en UCIN a identificar los indicadores que mejor predicen la aparición del diagnóstico en cuestión. De este modo, contribuirá a una inferencia más precisa, orientando la selección y la implementación de intervenciones adecuadas para la asistencia de enfermería de esta población, reforzando la relevancia de la investigación.

Ante esta problemática, se plantearon las siguientes preguntas: ¿Cuál es la prevalencia de la DE por localización adversa del catéter central de inserción periférica en neonatos ingresados en la UCIN? ¿Cuál es la precisión de sus indicadores clínicos?

Por lo tanto, el objetivo del presente estudio fue determinar las medidas de precisión de los indicadores clínicos de la DE de la CIPE^®^ por localización adversa del catéter central de inserción periférica en neonatos ingresados en la UCIN.

## MÉTODO

### Diseño del Estudio

La presente investigación se refiere a un fragmento de un proyecto más amplio, cuyo objetivo fue validar clínicamente los indicadores clínicos de los diagnósticos de enfermería de un subconjunto terminológico de la CIPE^®^ para recién nacidos con CCIP en una UCIN^(8,9)^. A partir de su uso en la práctica clínica, se identificó la necesidad de actualizarlo y mapearlo con la *Systematized Nomenclature of Medicine Clinical Terms* (SNOMED CT). En este sentido, se llevó a cabo una nueva validación del contenido por parte de especialistas para su posterior validación clínica mediante la precisión diagnóstica.

Teniendo en cuenta el gran número de diagnósticos presentes en dicho subconjunto, en este manuscrito se ha optado por presentar únicamente la precisión del DE de localización adversa del catéter central de inserción periférica, ya que fue el más prevalente durante la validación clínica. Por lo tanto, se trata de un estudio de precisión diagnóstica, con un diseño analítico y un enfoque cuantitativo. El informe se elaboró siguiendo las directrices de la iniciativa *Standards for Reporting Studies of Diagnostic Accuracy* (STARD), garantizando la claridad, la transparencia y la calidad en la presentación de los resultados^(11)^.

Se midieron los indicadores clínicos del DE mencionado, incluyendo la dificultad de progresión del catéter, la ausencia de retorno sanguíneo, el lugar de inserción del catéter, la prematuridad y la ocurrencia de eventos adversos, de acuerdo con las siguientes definiciones operativas:

- Dificultad para avanzar el catéter: caracterizada por la incapacidad de avanzar el catéter a través del introductor o la circulación periférica, por la imposibilidad de alcanzar la distancia previamente medida o por la dificultad para pasar el catéter de la circulación periférica a la circulación central;
- Ausencia de retorno sanguíneo: se produce cuando el insertador del catéter no obtiene retorno del flujo sanguíneo inmediatamente después de la inserción;
- Lugar de inserción del catéter: se refiere al sitio elegido por el insertador para la introducción y el avance del catéter;
- Prematuridad: definida como el nacimiento de un recién nacido antes de completar la semana 37 de gestación, es decir, con una edad gestacional inferior a 37 semanas;
- Ocurrencia de eventos adversos: complicaciones que pueden surgir durante o después del uso del catéter, comprometiendo la seguridad y eficacia del tratamiento intravenoso^(12,13)^.

### Lugar

El estudio se llevó a cabo en una UCIN del noreste de Brasil, vinculada al Sistema Único de Salud.

### Población

La población del estudio estuvo compuesta por 40 neonatos ingresados en la UCIN y en uso de CCIP en una maternidad de cuidados críticos del noreste de Brasil. Además de los recién nacidos, otros participantes formaron parte del estudio, entre ellos cuatro enfermeros que actuaron como evaluadores de los estudios de casos.

### Criterios de Selección

La selección de los recién nacidos se realizó por conveniencia de forma consecutiva, adoptando los siguientes criterios de inclusión: recién nacidos ingresados en la UTIN en uso de CCIP y que permanecieran en el sector hasta el final del tratamiento. Los criterios de exclusión adoptados fueron: traslado del recién nacido a otra institución y recién nacidos cuyas madres eran adolescentes. La exclusión de madres adolescentes se basó en las normas éticas que rigen la investigación con seres humanos (Resolución CNS 466/12), ya que los menores de edad, incluidos los adolescentes, no tienen plena capacidad civil y, por lo tanto, necesitan un responsable legal para firmar el Término de Consentimiento Libre y Esclarecido (TCLE). Cabe destacar que el TCLE fue leído y explicado, y que se aclararon todas las dudas a los padres o responsables del recién nacido.

En cuanto a la selección de los enfermeros, estos contaban con conocimientos y experiencia en terapia intensiva neonatal y son insertadores de catéteres CCIP, además de cumplir todos los criterios de inclusión. Los criterios de inclusión fueron: tener especialidad en neonatología y experiencia clínica asistencial de al menos cinco años en el área; tener un curso de habilitación en inserción de catéteres; tener experiencia práctica igual o superior a dos años en la inserción de CCIP. Los profesionales ausentes por licencia o vacaciones durante el período de recolección de datos fueron considerados criterios de exclusión.

El acceso a los enfermeros especialistas se realizó de forma presencial en el hospital donde se llevó a cabo la investigación. Durante el contacto inicial, se les invitó a participar y se les explicaron los objetivos del estudio, así como los riesgos y beneficios que conllevaba. Posteriormente, aquellos que aceptaron participar firmaron el TCLE.

### Definición de la Muestra

Para establecer la muestra de recién nacidos, se utilizó la fórmula de cálculo de muestras para poblaciones finitas, con el fin de proporcionar de manera imparcial, segura y científica la eficiencia y eficacia de esta investigación. El tamaño de la muestra se calculó siguiendo el siguiente procedimiento: se asignó un nivel de significación (a) del 5% y un margen de error del 0,05. Cuando la proporción era desconocida, se adoptó p = 0,5, considerando la varianza máxima. Para la selección de los especialistas del estudio, se utilizó un muestreo intencional no probabilístico.

### Recolección de Datos

Para alcanzar el objetivo, el proyecto global se llevó a cabo de acuerdo con las siguientes fases: 1) Implementación del proceso de enfermería de acuerdo con las etapas: evaluación de enfermería, DE, planificación de enfermería, implementación y evolución de la enfermería; 2) Elaboración de los estudios de casos y enunciados de los diagnósticos/resultados e intervenciones de enfermería (DE/RE/IE); 3) Análisis de concordancia entre los enfermeros especialistas; 4) Precisión de los indicadores clínicos de los diagnósticos de enfermería del subconjunto terminológico^(8)^. El período de recolección de datos fue de octubre a diciembre de 2024.

Los enunciados de los DE y sus respectivos indicadores clínicos se probaron en la población de neonatos, utilizando las siguientes medidas de precisión: sensibilidad, especificidad, valor predictivo positivo y negativo, razón de verosimilitud positiva y negativa.

Los datos de los pacientes se recopilaron mediante examen físico y análisis de los registros de enfermería contenidos en los historiales de la muestra. La recolección de datos y la inferencia diagnóstica fueron realizadas por la investigadora principal, junto con una estudiante del curso de doctorado en Enfermería.

Para el estudio, se utilizó un instrumento basado en el modelo teórico de Betty Neuman, estructurado en las siguientes secciones: datos sociodemográficos, evaluación, diagnósticos, planificación, implementación y evolución de la enfermería. Antes de la aplicación definitiva, se realizó una prueba piloto, previamente aprobada por el comité de ética en investigación, con el objetivo de verificar la idoneidad del instrumento. Cabe destacar que los participantes en la prueba piloto no fueron incluidos en el estudio principal.

Los especialistas recibieron hojas de cálculo elaboradas por el dúo, cada una correspondiente a un paciente y conteniendo todos los términos y DE/RE e IE de acuerdo con el modelo teórico empleado. Las hojas de cálculo también incluían información complementaria sobre datos socioeconómicos, clínicos y observaciones relevantes para el proceso de inferencia diagnóstica. Cada uno evaluó por separado la presencia o ausencia de DE, RE e IE en cada una de las hojas de cálculo recibidas.

Para esta investigación, se consideraron clínicamente validados los DE/RE e IE identificados en recién nacidos, descritos en los estudios de caso e incluidos en el subconjunto terminológico. El análisis de concordancia entre los enfermeros jueces se realizó mediante el coeficiente de concordancia de Kappa (k), y los especialistas analizaron los estudios de caso y los enunciados enumerados.

La última etapa del estudio consistió en evaluar la precisión de los indicadores clínicos de los DE del subconjunto terminológico, con el objetivo de identificar la exactitud de estos indicadores en la población de neonatos en uso de CCIP. Así, se analizó qué indicador presentaba mayor precisión en la predicción de la ocurrencia de los DE investigados.

En el presente estudio, los indicadores clínicos se definieron basándose en la literatura, que respalda la toma de decisiones clínicas. Teniendo en cuenta la limitación del número de páginas de la revista, este artículo se centrará exclusivamente en la precisión de los indicadores clínicos del DE de la CIPE «localización adversa del catéter central de inserción periférica», ya que fue el más prevalente en la investigación. Este enfoque es necesario debido a la gran cantidad de indicadores de DE que componen el subconjunto. Cabe destacar que otros resultados del proyecto global se publicarán en futuras producciones científicas.

### Análisis y Tratamiento de los Datos

El análisis de los datos se realizó mediante la verificación del nivel de concordancia y el nivel de consistencia (fiabilidad) de la opinión de los jueces (enfermeros especialistas) con respecto a los demás ítems del subconjunto terminológico, aplicando el coeficiente de concordancia de Kappa (k)^(14)^.

Este índice se aplicó también para verificar la concordancia entre los evaluadores en relación con cada elemento. El coeficiente de concordancia de Kappa evalúa la proporción de concordancia, que varía de «menos 1» a «más 1»; cuanto más se aproxima a 1, mejor es el nivel de concordancia entre los observadores. Cada juez indicó si el ítem era relevante y si las respectivas definiciones eran claras y precisas, eligiendo una opción entre las cinco respuestas sugeridas (totalmente de acuerdo – 1; parcialmente de acuerdo – 2; indiferente – 3; parcialmente en desacuerdo – 4; totalmente en desacuerdo – 5).

A cada opción se le asignó una puntuación: totalmente de acuerdo – 1,0; parcialmente de acuerdo – 0,75; indiferente – 0,50; parcialmente en desacuerdo – 0,25; totalmente en desacuerdo – 0,0. Este criterio se utilizó tanto para la pertinencia como para la aplicabilidad de cada ítem. Como criterio de aceptación, se estableció una concordancia ≥ 0,80 entre los jueces, lo que se consideró un buen nivel.

La concordancia entre los cuatro enfermeros especialistas se evaluó mediante el coeficiente Kappa (k), clasificado de la siguiente manera: pobre (de 0 a 0,19); relativa (de 0,20 a 0,39); moderada (de 0,40 a 0,59); sustancial (de 0,60 a 0,79); casi perfecta (de 0,80 a 0,99); y perfecta (igual a 1)^(15)^.

Se utilizó la prueba no paramétrica de Kruskal-Wallis para verificar si existían diferencias en las medias de las evaluaciones de los enfermeros especialistas. Se adoptó un nivel de significación de p ≤ 0,05; así, una diferencia entre las medias se consideró estadísticamente significativa cuando el valor de p era menor o igual a ese límite. Para evaluar la fiabilidad y aplicabilidad del subconjunto en diferentes contextos, se utilizó el coeficiente de correlación intraclase (CCI), donde CCI ≥ 0,75 indica una fiabilidad excelente; CCI entre 0,40 y 0,75 denota una fiabilidad satisfactoria; y CCI < 0,40 se considera pobre^(14,15)^.

Los datos relativos al DE y RE se analizaron mediante estadísticas descriptivas, teniendo en cuenta las frecuencias absolutas y relativas. La información sobre la IE se presentó de forma agrupada, siguiendo las categorías del modelo teórico, y se discutió según sus frecuencias. Posteriormente, se organizaron en una hoja de cálculo y, tras una doble verificación de la transcripción, se exportaron al *Statistical Package for Social Science* (SPSS), versión 20.0 Windows. Tras la codificación y tabulación, los datos se analizaron mediante lectura reflexiva y estadística, con la aplicación de la prueba de Kappa utilizando la Online Kappa Calculator.

Basándose en los objetivos del estudio y en su naturaleza, se formularon las siguientes hipótesis: Hipótesis nula (H_0_): Los indicadores clínicos de DE por localización adversa del catéter central de inserción periférica no presentan medidas de precisión significativas para la inferencia diagnóstica en neonatos ingresados en la UCIN. Hipótesis alternativa (H_1_): Los indicadores clínicos de DE por localización adversa del catéter central de inserción periférica presentan medidas de precisión significativas para la inferencia diagnóstica en neonatos ingresados en la UCIN.

El análisis de la validación clínica se llevó a cabo en dos fases. La primera fase consistió en la caracterización de la población, utilizando estadística descriptiva. En la segunda fase, se examinaron las medidas de precisión de los indicadores clínicos en relación con DE. Estas medidas se calcularon basándose en la inferencia diagnóstica de la presencia de DE, estableciendo un punto de corte del 50%. Para evaluar las asociaciones entre las variables cualitativas y los indicadores clínicos, se aplicó la prueba de chi cuadrado o la prueba exacta de Fischer.

### Aspectos Éticos

El estudio fue aprobado por el Comité de Ética en Investigación con el dictamen número 7.171.001 de 2024 y cumplió con los principios éticos de la Resolución n.º 466, de 12 de diciembre de 2012, del Consejo Nacional de Salud, que trata sobre la investigación con seres humanos.

## RESULTADOS

Participaron en este estudio 40 recién nacidos, de los cuales el 65% (26) eran mujeres, con predominio de bebés nacidos por cesárea (70%), bajo peso al nacer (65%) y con una media de APGAR de 6,46 y 8,52 en el primer y quinto minuto de vida, respectivamente. La estancia media en la UCIN fue de 10,23 días.

Las principales indicaciones para la inserción del catéter fueron la antibioterapia (80%) y la nutrición parenteral (65%). En cuanto al diagnóstico clínico, las afecciones respiratorias (75%) y la prematuridad (70%) fueron las más prevalentes. El tipo de catéter utilizado fue de silicona y de un solo lumen. En cuanto a los sitios de inserción, los miembros superiores fueron los más prevalentes (55%). Todos los catéteres fueron insertados por enfermeros.

En relación con la prevalencia de los indicadores clínicos de la DE de la CIPE^®^, localización adversa del catéter central de inserción periférica, se observó: «dificultad de progresión del catéter» (57,5%), «ausencia de retorno sanguíneo» (55%), «lugar de inserción del catéter (miembros superiores)» (55%), «ocurrencia de eventos adversos» (50%), «prematuridad (< 37 semanas)» (47,5%), «peso del recién nacido» (45%), «presencia de catéter venoso umbilical» (32,5%), «presencia de otro catéter de inserción periférica» (27,5%), «malformación congénita» (7,5%).

La Tabla 1 presenta las razones de prevalencia de los indicadores clínicos analizados según la ocurrencia de DE de localización adversa del catéter central de inserción periférica en neonatos ingresados en la UTIN. Los datos evidencian los indicadores clínicos que presentaron una asociación estadísticamente significativa con el diagnóstico, así como sus razones de prevalencia y intervalos de confianza. Estos hallazgos destacan los factores más relevantes para la inferencia diagnóstica, contribuyendo a un análisis más preciso y fundamentado.

**Tabla 1:** Razones de prevalencia de los indicadores clínicos según la ocurrencia de DE por localización adversa del catéter central de inserción periférica en recién nacidos en UTIN – Natal, RN, Brasil, 2024.

Variables	DE localización adversa del catéter cen-tral de inserción periférica						
Indicadores clínicos	Presente		Ausente		Total		Estadísticas
	N	%	N	%	N	%	
Dificultad en la progresión del catéter							
Presente	23,0	57,5	0,0	0,0	23,0	57,5	*p* = 0,001*
Ausente	0,0	0,0	17,0	42,5	17,0	42,5	RP^†^ = 4,98^†^
Total	23,0	57,5	17,0	42,5	40,0	100,0	IC^‡^95%: 3,09–5,88^‡^
Falta de retorno sanguíneo							
Presente	21,0	52,5	1,0	2,5	22,0	55,0	*p* = 0,001*
Ausente	4,0	10,0	14,0	35,0	18,0	45,0	RP^†^ = 4,02^†^
Total	23,0	57,5	17,0	42,5	40,0	100,0	IC^‡^95%: 2,71–5,17^‡^
Lugar de inserción del catéter (miembros superiores)							
Presente	19,0	47,5	3,0	7,5	22,0	55,0	*p* = 0,038*
Ausente	4,0	10,0	14,0	35,0	18,0	45,0	RP^†^ = 3,95^†^
Total	23,0	57,5	17,0	42,5	40,0	100,0	IC^‡^95%: 2,15–4,80^‡^
Acontecimientos adversos							
Presente	18,0	45,0	2,0	5,0	20,0	50,0	*p* = 0,002*
Ausente	5,0	12,5	15,0	37,5	20,0	50,0	RP^†^ = 2,98^†^
Total	23,00	57,5	17,0	42,5	40,0	100,0	IC^‡^95%: 1,84–3,56^‡^
Prematuridad (< 37 semanas)							
Presente	16,0	40,0	3,0	7,5	19,0	47,5	*p* = 0,012*
Ausente	7,0	17,5	14,0	35,0	21,0	52,5	RP^†^ = 2,92^†^
Total	23,0	57,5	17,0	42,5	40,0	100,0	IC^‡^95%: 1,69–3,28^‡^
Peso del recién nacido							
Presente	11,0	27,5	7,0	17,5	18,0	45,0	*p* = 0,332§
Ausente	12,0	30,0	10,0	25,0	22,0	55,0	RP^†^ = 1,48^†^
Total	23,0	57,5	17,0	42,5	40,0	100,0	IC^‡^95%: 0,86–1,98^‡^
Presencia de catéter venoso umbilical							
Presente	9,0	22,5	4,0	10,0	13,0	32,5	*p* = 0,217*
Ausente	14,0	35,0	13,0	32,5	27,0	67,5	RP^†^ = 1,46^†^
Total	23,0	57,5	17,0	42,5	40,0	100,0	IC^‡^95%: 0,71–1,92^‡^
Presencia de otro catéter venoso de inserción periférica							
Presente	8,0	20,0	3,0	7,5	11,0	27,5	*p* = 0,402*
Ausente	15,0	37,5	14,0	35,0	29,0	72,5	RP^†^ = 1,34^†^
Total	23,0	57,5	17,0	42,5	40,0	100,0	IC^‡^95%: 0,97–2,28^‡^
Malformación congénita							
Presente	2,0	5,0	1,0	2,5	3,0	7,5	*p* = 0,619*
Ausente	21,0	52,5	16,0	40,0	37,0	92,5	RP^†^ = 1,21^†^
Total	23,0	57,5	17,0	42,5	40,0	100,0	IC^‡^95%: 0,82–2,12^‡^

*Prueba exacta de Fisher;

^†^PR = razón de prevalencia;

^‡^IC = intervalo de confianza del 95%;

^§^prueba de chi cuadrado de Pearson; p < 0,05.

Según la Tabla 1, los indicadores clínicos “Dificultad en la progresión del catéter”, “Ausencia de retorno sanguíneo”, “Lugar de inserción del catéter (miembros superiores)”, “Aparición de eventos adversos” y “Prematuridad (< 37 semanas)” mostraron una relación de riesgo con el diagnóstico de enfermería (DE) estudiado. Además, se observó un aumento de la probabilidad de que desarrollaran el DE localización adversa del catéter central de inserción periférica, según las siguientes razones de prevalencia (RP): 5 veces (RP = 4,98), 4 veces (RP = 4,02), 4 veces (RP = 3,95), 3 veces (RP = 2,98), 3 veces (RP = 2,92), respectivamente.

Por otro lado, los indicadores “Peso del recién nacido”, “Presencia de un catéter venoso umbilical”, “Presencia de otro catéter de inserción periférica” y “Malformación congénita” no mostraron una asociación estadísticamente significativa con la localización adversa en urgencias del catéter central de inserción periférica.

En la Tabla 2 se detallan las medidas de precisión de los indicadores clínicos relacionados con la localización adversa en urgencias del catéter central de inserción periférica en los recién nacidos ingresados en la Unidad de Cuidados Intensivos Neonatales.

**Tabla 2:** Medidas de precisión de los indicadores clínicos del DE, localización adversa del catéter central de inserción periférica en recién nacidos en la Unidad de Cuidados Intensivos Neonatales – Natal, RN, Brasil, 2024.

Indicadores clínicos	Se*	Es^†^	VPP^‡^	VPN^§^	RVP^||^ (IC 95%)	NNR^¶^ (IC 95%)	ORD** (IC 95%)
Dificultad en la progresión del catéter	100,0	45,28	100,0	16,97	9,00 (7,87–11,67)	0,67 (0,31–0,91)	8,92 (6,91–10,68)
Falta de retorno sanguíneo	98,34	51,10	98,41	29,55	6,31 (4,24–8,12)	0,62 (0,25–0,87)	6,21 (4,16–8,74)
Lugar de inserción del catéter (extremidades superiores)	57,31	92,30	97,12	23,45	4,84 (2,14–6,92)	0,53 (0,41–0,77)	5,00 (3,24–7,61)
Acontecimientos adversos	48,87	89,93	91,39	19,38	3,33 (1,18–5,73)	0,30 (0,19–0,64)	4,93 (2,84–6,75)
Prematuridad (< 37 semanas)	54,21	94,85	99,82	22,31	3,46 (1,28–5,98)	0,76 (0,62–0,83)	6,90 (4,55–8,65)
Peso del recién nacido	13,26	54,58	97,50	28,44	1,26 (0,02–2,63)	0,88 (0,53–1,69)	2,76 (0,95–4,08)
Presencia de catéter venoso umbilical	31,16	64,07	57,41	26,84	1,46 (0,65–3,86)	0,65 (0,21–1,10)	1,93 (0,89–4,32)
Presencia de otro catéter venoso de inserción periférica	33,08	64,13	96,30	22,17	1,98 (0,85–4,12)	0,82 (0,55–1,17)	2,11 (0,91–4,85)
Malformación congénita	3,29	44,55	97,50	18,42	1,37 (0,14–3,29)	0,55 (0,13–1,12)	1,41 (0,67–3,28)

*Se = sensibilidad;

^†^Es = especificidad;

^‡^VPP = valor predictivo positivo;

^§^VPN = valor predictivo negativo;

^||^RVP = razón de verosimilitud positiva;

^¶^RVN = *razón de*verosimilitud negativa;

**ORD = *Odds Ratio*diagnosis.

La prevalencia del DE localización adversa del catéter central insertado periféricamente fue del 57,5%. Los indicadores clínicos “dificultad en la progresión del catéter” y “ausencia de retorno sanguíneo” presentaron valores de especificidad elevados, así como cocientes de probabilidad y ORD estadísticamente significativos, lo que sugiere que son buenos indicadores para confirmar la presencia del diagnóstico.

Además, los indicadores clínicos “lugar de inserción del catéter”, “prematuridad” y “aparición de acontecimientos adversos” mostraron valores de sensibilidad más altos, acompañados de valores de probabilidad y ORD estadísticamente significativos, lo que indica que estos indicadores tienen las mejores medidas de precisión para inferir el DE estudiado.

## DISCUSIÓN

La ubicación adversa del catéter central de inserción periférica fue validada clínicamente mediante medidas de precisión, lo que corrobora la literatura, que enfatiza la importancia de vigilar la posición de la punta del catéter, ya que la incidencia de una mala posición varía entre el 10% y el 60%. Esta inadecuación puede dar lugar a eventos adversos significativos, como taponamiento cardíaco, derrame pleural y pericárdico, infiltración, lesión endotelial, entre otros, lo que representa un riesgo considerable para la seguridad del paciente^(16–18)^.

Se considera que la ocurrencia de una colocación inadecuada del catéter CCIP en recién nacidos bajo cuidados intensivos es significativamente más común, estimándose en aproximadamente tres veces mayor en comparación con otros accesos centrales^(19)^.

Se ha informado que la migración del CCIP ocurre en las primeras 24–72 horas después de la inserción^(12,20)^. Por lo tanto, algunos investigadores han sugerido repetir la radiografía 24 horas después de la inserción de un CCIP para identificar la migración, mientras que otros han sugerido realizar una vigilancia por imagen hasta 72 horas después de la inserción del CCIP^(12)^.

En cuanto a los factores de riesgo, el indicador clínico «dificultad de progresión del catéter» se consideró un predictor del DE en estudio. Este hallazgo puede evidenciarse en la medida en que la progresión inadecuada se produce debido a la trayectoria incorrecta del CCIP, lo que da lugar a un enredamiento del catéter y a su avance hacia los vasos colaterales próximos a la circulación central (venas subclavia, yugular y axilar), lo que se asocia al desenlace de posición periférica^(21)^. La dificultad de progresión del CCIP puede definirse como la incapacidad de pasar el catéter por el introductor o por la circulación periférica, la incapacidad de pasar el catéter hasta la distancia previamente medida o la incapacidad de pasar el catéter de la circulación periférica a la circulación central^(12)^.

En un estudio realizado en una UCIN, se observó dificultad en la progresión en 63 procedimientos (25,8%), lo que indica una correlación significativa con la posición inicial de la punta del catéter. La dificultad de progresión se notificó en el 50% de los catéteres con posición inicial no central y en el 22% de los catéteres con posición central^(22)^. Otro estudio que corrobora los hallazgos de la presente investigación se realizó con 141 neonatos, en el que se identificó que la dificultad de progresión del CCIP durante la inserción se asoció con la migración de la punta del catéter (p < 0,01)^(12)^.

La «ausencia de retorno sanguíneo» inmediatamente después de la inserción del catéter, también llamada prueba de flujo y reflujo, se consideró un factor de riesgo para la trayectoria falsa del CCIP y fue un indicador clínico relacionado con la DE estudiada, considerándose también un predictor de la localización adversa del catéter central de inserción periférica en neonatología. Este hallazgo corrobora la literatura, que destaca que, además de la dificultad en el avance del catéter durante la inserción, la aspiración sanguínea deficiente se ha descrito como un factor predictivo de que pueda haberse producido una mala implantación del catéter^(23)^.

Una investigación que involucró a 100 recién nacidos indicó que la probabilidad de que se produzcan eventos como la obstrucción del catéter cuando la punta del catéter se encuentra en una región periférica es hasta siete veces mayor que cuando se encuentra en posición central, con el consiguiente perjuicio en el retorno sanguíneo durante la aspiración^(24)^.

El «lugar de inserción del catéter» fue identificado por los diagnosticadores como indicador clínico, pero no se consideró un factor predictivo de la DE. Un estudio que evaluó 1234 catéteres colocados en 918 bebés hospitalizados demostró que la mala colocación en las primeras 72 horas y a lo largo del tiempo fue más frecuente en los catéteres de los miembros superiores^(4)^.

Según un estudio realizado en China, en comparación con las venas basilares, temporales superficiales o femorales, se observó, tras el análisis de 40 pacientes sometidos a cateterismo, que el CCIP, cuando se accede a través de la vena femoral, puede mejorar la tasa de éxito del cateterismo, acortar el tiempo de inserción y reducir las complicaciones, como la flebitis mecánica, debido a la fricción, ya que los vasos sanguíneos de las extremidades inferiores son más gruesos y profundos^(25)^.

En este estudio se observó una alta frecuencia del indicador clínico «prematuridad», dato que también se encontró en otros estudios, donde el uso de CCIP en recién nacidos ingresados en la UTI presenta una mayor prevalencia en neonatos prematuros. La frecuencia del uso de CCIP en recién nacidos prematuros está relacionada con la predisposición a complicaciones debido a la inmadurez de los órganos y sistemas, el bajo peso y el alto riesgo de mortalidad, lo que los hace necesitar una terapia intravenosa segura^(26)^.

La prematuridad (edad gestacional al nacer <37 semanas) se ha asociado a un mayor riesgo de diversas complicaciones, entre ellas la mala colocación, además de eventos adversos como flebitis, eritema, edema y alteraciones de la perfusión^(4)^.

Estos eventos adversos también representan un factor predisponente para la retirada precoz del catéter. Un estudio realizado con 216 neonatos en uso de CCIP, la mayoría prematuros, señaló que la retirada precoz del catéter se produjo, en promedio, 8 días después de la inserción. Esta retirada se asoció a la dificultad de mantenimiento, lo que provocó obstrucción, rotura, flebitis y extrusión. Además, el estudio indicó que el uso de múltiples antibióticos, junto con el plan de nutrición parenteral, está asociado al bajo peso de la población neonatal, lo que constituye un factor causal relevante para la retirada anticipada del dispositivo^(27)^.

La prematuridad también se ha demostrado como un factor de riesgo para la retirada del CCIP por sospecha de infección. El riesgo de infección es más frecuente en neonatos con mayor gravedad clínica, lo que requiere cambios más frecuentes de la terapia de infusión debido a su inestabilidad, lo que da lugar a una mayor manipulación del catéter^(28)^.

El indicador clínico «ocurrencia de eventos adversos» fue identificado como predictivo de la DE de localización adversa del catéter central de inserción periférica por los diagnosticadores. Un ensayo clínico aleatorizado observó una asociación entre la localización final de la punta del CCIP y la incidencia de eventos adversos. En los procedimientos de CCIP en los que la punta estaba mal posicionada, se observó que el ODR para la ocurrencia de eventos adversos fue 4,10 veces mayor en comparación con los procedimientos en los que la punta estaba posicionada centralmente^(21)^.

La trombosis, la embolia, las lesiones vasculares, la obstrucción del catéter y el desplazamiento son complicaciones potenciales asociadas a la retirada no planificada de los catéteres. Entre ellas, las infecciones representan una preocupación significativa, ya que el acceso directo al torrente sanguíneo y la manipulación frecuente aumentan el riesgo de colonización bacteriana. Estudios de revisión señalan la asociación entre la retirada precoz del catéter por sospecha de infección y factores como la prematuridad, el bajo peso, la posición no central de la punta del catéter y el uso prolongado del CCIP^(29)^.

Un estudio reveló que las variables asociadas a la prevalencia de complicaciones durante el uso del CCIP incluyen: sexo masculino, bajo peso, edad superior a 48 horas de vida, más de tres intentos de inserción del CCIP, complicaciones durante la inserción, cambios frecuentes de vendajes, dificultad para el avance del catéter y ubicación no central del dispositivo. Por lo tanto, la inserción y el mantenimiento del CCIP por parte de enfermeras capacitadas es esencial para la identificación temprana de signos de complicaciones, lo que contribuye a la seguridad en el uso del dispositivo en neonatos^(30)^.

El indicador clínico «peso del recién nacido» no fue considerado por los diagnosticadores como un predictor de DE por localización adversa del catéter central de inserción periférica. Sin embargo, se observó que cuanto menor era el peso del recién nacido, mayor era la probabilidad de que se produjera este diagnóstico. En la institución donde se realizó la investigación, el protocolo de medición para la inserción del catéter tiene en cuenta la medida anatómica superficial, que comprende la distancia desde el lugar de punción hasta el extremo externo de la clavícula, llegando al tercer espacio intercostal.

Un estudio cuyo objetivo era desarrollar diagramas y tablas para recomendar las profundidades de inserción del CCIP en neonatos, basándose en parámetros antropométricos, demostró que las tablas desarrolladas permitían determinar de forma rápida y precisa las profundidades de inserción recomendadas del CCIP en neonatos^(6)^.

Los estudios que exploraron la relación entre las medidas antropométricas neonatales y la profundidad adecuada de inserción del CCIP demostraron una correlación entre el peso y la longitud del recién nacido, lo que sugiere que estas variables pueden servir como modelo antropométrico para determinar la profundidad de inserción del CCIP neonatal^(23,31)^.

Se observó que el método modificado de medición del CCIP dio lugar a una mayor incidencia de localización central y a una menor incidencia de localización intracardíaca, en comparación con el método tradicional. En el grupo que utilizó la medida tradicional, la frecuencia de posicionamiento central fue del 2,3%, mientras que la localización intracardíaca se produjo en el 72,7% de los casos^(23)^.

La DE de localización adversa del catéter venoso central de inserción periférica en unidades neonatales refuerza la importancia de identificar medidas que minimicen los errores en la localización del catéter y de adoptar intervenciones de enfermería orientadas a la prevención del DE estudiado.

Los hallazgos de este estudio tienen implicaciones significativas para la práctica clínica de enfermería, ya que contribuyen a la inferencia diagnóstica y a la formulación de intervenciones más precisas, lo que favorece resultados positivos y sensibles en la atención neonatal.

La identificación precisa de los indicadores clínicos ayuda a prevenir complicaciones relacionadas con el uso del CCIP, promoviendo la seguridad, la reducción de las infecciones del torrente sanguíneo y la minimización del malestar neonatal. Además, los hallazgos refuerzan el papel esencial del enfermero en la UCIN, cuya actuación cualificada y basada en la evidencia permite la identificación precoz de complicaciones, la implementación de protocolos seguros y la garantía de la calidad de la atención al recién nacido.

Por último, la realización del estudio en un solo servicio constituye una limitación, ya que restringe el análisis a una única realidad institucional, reflejando prácticas, protocolos y especificidades asistenciales que pueden comprometer la generalización de los hallazgos. Por lo tanto, se recomienda la realización de estudios multicéntricos, que amplíen la diversidad de escenarios y permitan la aplicabilidad externa de las evidencias producidas.

## CONCLUSIÓN

Los resultados del presente estudio confirmaron la hipótesis alternativa al demostrar que los indicadores clínicos de DE localización adversa del catéter central de inserción periférica presentaron medidas de precisión significativas. Estos hallazgos evidencian la fiabilidad y precisión de estos indicadores para la inferencia diagnóstica en neonatos ingresados en unidades de terapia intensiva neonatal. Los indicadores clínicos «dificultad para avanzar el catéter» y «ausencia de retorno sanguíneo» destacaron por sus elevados valores de especificidad, mientras que «lugar de inserción del catéter», «prematuridad» y «ocurrencia de eventos adversos» presentaron una mayor sensibilidad, lo que refuerza la validez de la inferencia diagnóstica basada en estos factores.

El estudio enfatiza la necesidad de validar clínicamente los diagnósticos de enfermería de la CIPE® en la UCIN, especialmente en relación con los demás enunciados del subconjunto terminológico. La continuidad de esta investigación puede contribuir a mejorar las taxonomías de enfermería, fortaleciendo la toma de decisiones clínicas y cualificando la asistencia al recién nacido crítico.

Además, los hallazgos podrían servir de base para protocolos de asistencia basados en la evidencia, fortalecer el lenguaje estandarizado en enfermería y fomentar la investigación multicéntrica, ampliando la aplicabilidad de los resultados y su impacto en la seguridad del paciente neonatal.

## DISPONIBILIDAD DE DATOS

Los datos utilizados durante este estudio están disponibles del autor correspondiente previa solicitud razonable.
